# Kinetics of Seawater Diffusion into an Anticorrosion Coating with Modified Carbon Nanomaterials

**DOI:** 10.3390/nano16211351

**Published:** 2026-10-24

**Authors:** Sergei V. Yakovlev, Stepan Yu. Kupreenko, Dmitrii N. Stolbov, Anton S. Ivanov, Andrey N. Kharlanov, Konstantin I. Maslakov, Evgeniya V. Suslova, Serguei V. Savilov

**Affiliations:** 1 N. S. Kurnakov Institute of General and Inorganic Chemistry, Russian Academy of Sciences, Leninskiy ave. 31, Moscow 119991, Russia; yakovlevserguei@rambler.ru; 2 Department of Chemistry, M. V. Lomonosov Moscow State University, Leninskie Gory 1, Bld. 3, Moscow 119991, Russia; stolbovdn@gmail.com (D.N.S.); anthon.s.ivanov@gmail.com (A.S.I.); kharlanovan@my.msu.ru (A.N.K.); suslovaev@my.msu.ru (E.V.S.)

**Keywords:** anticorrosion coating, seawater diffusion, electrochemical impedance spectroscopy, open-circuit potential, electrochemical noise, carbon nanomaterials

## Abstract

The influence of modified (oxidized and doped) carbon nanomaterials added to a PVC-based enamel on the kinetics of seawater diffusion into a protective coating has been studied. To this end, the feasibility of using diffuse reflectance infrared spectroscopy and open-circuit potential (OCP) noise measurements of a three-electrode cell for investigating water diffusion kinetics was examined. The applicability of these methods was validated by electrochemical impedance spectroscopy (EIS) measurements. OCP noise and trend line monitoring has potential for broad application due to its simplicity and low cost compared to EIS, as well as its capability for diagnostics of anticorrosion coatings at the earliest stages of water diffusion into the coating. Oxidized few-layer graphene nanoflakes exhibited outstanding barrier properties (low water capacity, low rate of water diffusion into the coating, the high coating resistance and low rate of coating resistance degradation) as components of anticorrosion coatings. This performance is attributed to a high density of oxygen-containing functional groups, which ensure homogeneous nanofiller dispersion and promote strong interfacial adhesion to the polymer matrix via hydrogen bonding.

## 1. Introduction

Carbon nanomaterials (CNMs) are widely used as fillers in anticorrosion coatings for metallic ship components due to their outstanding barrier properties and the feasibility of their chemical modification [1,2,3,4,5,6]. CNMs impede the penetration of water and air, which lead to the deterioration of metallic ship parts through electrochemical reactions. Nevertheless, seawater inevitably penetrates to the coating–metal interface through the pore system and various defects of the coating. This leads to metal degradation through the formation of various corrosion products at the metal–coating interface, swelling, and subsequent film delamination. It is therefore evident that the rate of water diffusion into the anticorrosion coating directly affects the long-term performance of the protective coating. Numerous methods have been developed for its measurement, which can be broadly classified into gravimetric [7], electrochemical [7], and optical [8] techniques.

All methods for measuring the diffusion rate *D* are based on solving the diffusion equation into a film on an impermeable substrate [8,9]:

(1) $$\frac{\Delta m\left(t\right)}{m_{\infty }}=1-\frac{8}{\pi^{2}}\sum_{n=0}^{\infty }\frac{1}{{\left(2n+1\right)}^{2}}\exp\left(-D{\left(2n+1\right)}^{2}\frac{\pi^{2}t}{4d^{2}}\right),$$

which defines the time dependence of the mass of water Δ*m*(*t*), that has penetrated into a film of thickness *d*, normalized to the equilibrium water uptake *m*_∞_. In the gravimetric method, the change in coating mass is measured directly by weighing films immersed in water for various times. The inaccuracy of this method is associated with film drying during transfer from water to the balance and the necessity of preliminarily removing free water from the coating surface, which introduces a time delay in the measurement.

Electrochemical impedance spectroscopy (EIS) [7,10,11,12,13,14,15,16,17] is no less popular for calculating the water diffusion rate in a coating, as it allows measurements to be performed directly in an electrochemical cell with a 3.5% sodium chloride solution simulating seawater. It is based on the increase in coating electrical capacitance upon water uptake. Typically, the Brasher–Kingsbury equation [18] is used to estimate the volume fraction of water φ in the film:

(2) $$\varphi =\frac{\log\left(C_{t}/C_{0}\right)}{\log\left(\varepsilon_{W}\right)},$$

where *C*_t_ and *C*_0_ are the coating capacitance at time *t* and at the initial time, respectively, and ε_W_ is the relative permittivity of water, equal to 80 at room temperature. Assuming a constant coating thickness during diffusion, the following equation can be derived from (2) [19]:

(3) $$\frac{\Delta m\left(t\right)}{m_{\infty }}=\frac{\ln\left(C_{t}/C_{0}\right)}{\ln\left(C_{\infty }/C_{0}\right)}$$

Comparison with gravimetric measurements has shown that for calculations it is better to use the capacitance value obtained at high frequencies (e.g., 100 kHz) [12]. This is because in impedance spectroscopy, mass transfer occurs at low frequencies, whereas charge transfer occurs at high frequencies [7].

Assuming no film swelling and a Fickian diffusion mechanism and combining Equations (1) and (3), the water diffusion rate *D* can be calculated. Both the series solution of the diffusion equation and its asymptote for the initial stage of mass change can be used [20]:

(4) $$\frac{\Delta m\left(t\right)}{m_{\infty }}\approx \frac{2}{d}\sqrt{\frac{Dt}{\pi }}$$

Diffusion may deviate from Fick’s law because the relaxation rate of the polymer network of the coating is not always substantially faster than the diffusion rate. In the general case, the asymptote at short diffusion times has the form [21]:

(5) $$\frac{\Delta m\left(t\right)}{m_{\infty }}\approx Kt^{p},$$

where *K* is a constant, and the exponent *p* = 0.5 for Fickian diffusion. For *p* ≤ 1, the diffusion rate is substantially faster than the polymer network relaxation, and polymer swelling occurs [14]. For *p* > 1, the diffusion rate equals the relaxation rate [14].

There are various methods for accounting for polymer network relaxation; for example, for calculating the diffusion rate in glassy polymers, the following equation is used [11,22]:

(6) $$\frac{\ln\left(C_{t}/C_{0}\right)}{\ln\left(C_{\infty }/C_{0}\right)}=m^{*}\left[1-\frac{8}{\pi^{2}}\sum_{n=0}^{\infty }\frac{1}{{\left(2n+1\right)}^{2}}\exp\left(-D{\left(2n+1\right)}^{2}\frac{\pi^{2}t}{4d^{2}}\right)\right]+\left(1-m^{*}\right)\left[1-exp\left(kt\right)\right],$$

where *k* is the polymer relaxation coefficient, and m^*^ is a measure of the deviation of the transport mechanism from ideal Fickian behavior (a number from 0 to 1). *m* = 1 for Fickian diffusion and 0 for anomalous diffusion.

It should be noted that the diffusion rate *D* depends on temperature according to the classical Arrhenius equation [7,11]:

(7) $$D=D_{0}exp\left(-\frac{E_{a}}{RT}\right),$$

where *D*_0_ is the pre-exponential factor, *R* is the universal gas constant, and *E*_a_ is the activation energy of the diffusion process (the energy barrier that a molecule must overcome to diffuse). It is also worth noting that at elevated temperatures, irreversible interactions between water and polymers may occur, as well as polymer chain scission and segment leaching during hygrothermal aging [7].

The errors of the Brasher–Kingsbury equation are due to film thickness (which may not be constant because of swelling), permittivity (not equal to ε_W_ when pores are only partially filled), and electrochemical reactions at the polymer/metal interface [7].

As water diffuses into the coating, the specific capacitance increases and the film resistance decreases, so that the time constant τ=RC remains constant upon immersion in water [14]. Therefore, it would seem that the film resistance could be used instead of capacitance to calculate the diffusion rate. However, in [12], principal component analysis was used to study the correlation of electrochemical parameters with gravimetric data. It was shown that the correlation is stronger with capacitive parameters than with resistance, but it is advisable to consider all parameters. For calculations using the Brasher–Kingsbury formula, it is recommended to use the effective capacitance value calculated by [23]:

(8) $$C_{eff}=Y^{\frac{1}{n}}R^{\frac{\left(1-n\right)}{n}},$$

where *R* is the coating resistance, and *Y* и *n* are parameters of the constant phase element, often introduced to describe the impedance of a circuit with a protective coating. Its impedance is defined by [12]:

(9) $$Z_{CPE}=\frac{1}{Y{\left(i2\pi f\right)}^{n}},$$

where *i* is the imaginary unit, and *f* is the frequency. The exponent *n* can take values from 0 to 1. At *n* = 1, the constant phase element is equivalent to a capacitance, and the influence of coating resistance on the effective capacitance is zero (according to formula (8)).

**Figure 1:**
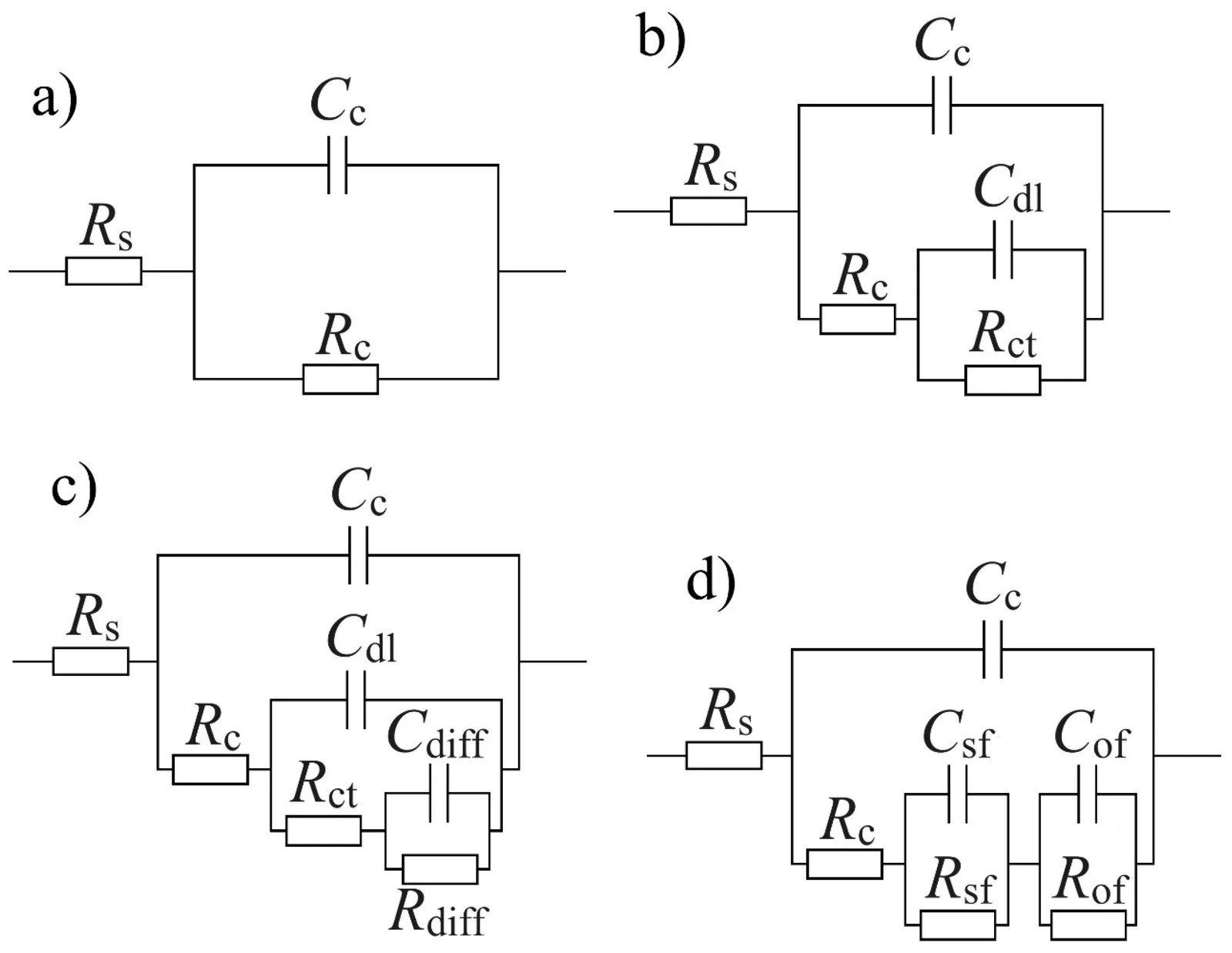
Equivalent circuit models for calculating the impedance of a protective coating on metal immersed in NaCl solution at (**a**) 16 min, (**b**) 24 h, (**c**) 47 h, and (**d**) 533 h, from [16].

Various equivalent circuit models have been proposed for evaluating the electrical parameters of a coating from the impedance curve. In [16], an analysis was performed and four impedance spectroscopy models were proposed to describe its behavior as water penetrates the protective coating and as it subsequently degrades. At the beginning of immersion, the equivalent electrical circuit consists of the solution resistance *R*_s_ and a parallel combination of the coating capacitance* C*_c_ (or, more generally, a constant phase element) and the coating resistance (sometimes called pore resistance) *R_c_* (Figure 1a). This model with a constant phase element is often used to describe the kinetics of water diffusion into a coating [11,12,14,15,16]. As water reaches the metal–coating interface, an electrochemical reaction occurs, requiring the introduction of additional impedances for the electrochemical reactions (Figure 1b): the double-layer capacitance *C_dl_* and the charge-transfer resistance (sometimes called polarization resistance) *Rct*. When the diffusion of corrosion products in the coatings is hindered, diffusion characteristics appear in the impedance model (Figure 1c): the diffusion constant phase element* C*_diff_ and the diffusion resistance *R*_diff_. Upon prolonged immersion, the formation of salt films containing chloride ions on the metal surface must be taken into account (Figure 1d): the salt film capacitance *C*_sf_, the salt film resistance *R*_sf_, the inner oxide film capacitance* C*_of_, and the inner oxide film resistance *R*_of_.

Coating failure can be identified by a drop in the low-frequency impedance to 10^6^ Ohm·cm^2^, and the quality of the barrier properties of coatings can be compared by the time required for the low-frequency impedance to decrease to a critical value, or by the diameter of the semicircle in the Nyquist plot at the same immersion time in NaCl solution, since it characterizes the dielectric properties of the coating [10].

In [11], the evolution of Nyquist plots and the parameters of the model in Figure 1b, which accounts for the formation of the electrical double layer, was shown for an epoxy coating on steel as a function of immersion time in 3.5% NaCl solution. The degree of coating delamination α (the ratio of the delaminated area to the total coating area) can be determined from the breakpoint frequency *f*_b_ of the impedance curve:

(10) $$f_{b}=\frac{1}{2\pi R_{c}C_{c}}=K\alpha ,$$

where $K=1/\left(2\pi \varepsilon \varepsilon_{0}\rho \right)$, which depends only on the coating parameters (relative permittivity ε and electrical resistivity ρ) [24]. The breakpoint frequency *f*_b_ is defined as the frequency at which the phase angle is 45° in the transition region from capacitive to resistive behavior [25]. However, the breakpoint frequency is not available if the coating resistance is high and/or the minimum phase angle in the high-frequency region exceeds 45°. The phase angle at 10 Hz θ_10 Hz_ is free from this drawback and reproduces the temporal evolution of the delamination degree α.

Optical methods for studying the kinetics of water diffusion into a protective coating are based on measuring optical properties (absorption, refractive index, fluorescence, etc.) [8]. Their disadvantage is the difficulty, and often even impossibility, of performing in situ measurements. For example, ellipsometry, a reliable method for measuring refractive indices and film thicknesses, is unsuitable because optical access to the coating immersed in water is impossible [8]. Neutron and X-ray reflectometry can measure only the diffusion of heavy water (D_2_O) vapor in situ [26,27,28]; measurements with the liquid phase are difficult. The method of large-angle interferometric refractometry has proven to be effective [8]: it allows simultaneous measurement of film thickness, average refractive index, and refractive index at the film–substrate interface every second, and from these, the water content in the coating and the water diffusion rate can be derived. Unfortunately, such setups are not commercially available.

Among optical methods for studying the kinetics of water diffusion into a protective coating, infrared spectroscopy occupies a special place [29,30]. It is based on measuring changes in the intensity of absorption bands corresponding to vibrations of water molecules. If a standard ATR (Attenuated Total Reflection) prism made of ZnSe and a clamping device are sufficient for measuring the kinetics of water diffusion into a free film [29], then for studying the polymer on an aluminum substrate, a Kretschmann configuration accessory is required [30]. In the Kretschmann cell, IR radiation impinging at an angle of 65° onto a hemispherical ZnSe crystal penetrates via an evanescent wave to a small depth through a thin aluminum sputtered layer into the polymer located in a flow cell and is absorbed, exciting vibrations of the polymer and water molecules. This design allows in situ observation of water diffusion kinetics and aluminum corrosion. For thick steel plates, ex situ measurements using a diamond ATR accessory or a diffuse reflectance accessory are more suitable [31]. When calculating the diffusion rate by IR spectroscopy, the solution of the diffusion equation in Equation (1) should be averaged over the depth from 0 to the penetration depth of the evanescent wave, taking advantage of its smallness compared to the coating thickness [9]. The final expression for describing the kinetics of water diffusion in a thin near-surface layer by IR spectroscopy is as follows:

(11) $$\frac{\Delta m\left(t\right)}{m_{\infty }}=1-\frac{4}{\pi }\sum_{n=0}^{\infty }\frac{{\left(-1\right)}^{n}}{2n+1}\exp\left(-D{\left(2n+1\right)}^{2}\frac{\pi^{2}t}{4d^{2}}\right),$$

As can be seen, there are numerous methods for studying the kinetics of water diffusion into an anticorrosion coating, each with its own advantages and disadvantages. The aim of this work is to study the effect of additives of modified (oxidized and heteroatom-doped) carbon nanomaterials on the kinetics of water diffusion into a protective coating using two new methods. In the first method, diffuse reflectance infrared spectroscopy is employed for the first time, and in the second, measurements of the open-circuit potential (OCP) noise of a three-electrode cell are proposed as an alternative to the widespread but expensive electrochemical impedance spectroscopy method. An advantage of the OCP noise method is also the possibility of diagnosing anticorrosion coatings with high resistance at the earliest stages of water diffusion, when EIS does not work because the input impedance of the potentiostat is lower than the coating resistance. The justification for the applicability of the methods was obtained using EIS.

A PVC-based enamel was selected as the coating material due to its high chemical resistance and durability against prolonged exposure to water, weathering and temperature fluctuations. PVC composites with carbon nanofillers have long attracted considerable attention [32]. In this study, oxidized carbon nanotubes (CNTox) and few-layer graphene nanoflakes (GNFox), as well as nitrogen- and phosphorus-doped GNFs (N-GNF and P-GNF), were chosen as fillers. The selection of these fillers is attributed to a number of factors.

Thus, pristine CNTs and GNFs possess outstanding barrier properties, as they are impermeable to liquids and gases; however, due to strong van der Waals interactions, they are prone to agglomeration (aggregation and restacking) [1,2]. Within the polymer matrix, this leads to the formation of defects and micropores, which, conversely, accelerate the penetration of water, oxygen, and aggressive chloride ions toward the metal substrate. Oxidation of carbon materials enhances wettability and chemical affinity for the polymer matrix or solvents used in PVC enamels, owing to polar functional groups on the surface [33]. This ensures uniform filler distribution without the formation of conglomerates. Well-dispersed oxidized materials create a highly tortuous barrier within the coating bulk, which significantly extends the diffusion path of water, oxygen, and chloride ions to the metal substrate. Oxygen-containing groups are capable of forming hydrogen bonds with the PVC polymer chain [33], thereby improving the mechanical integrity of the coating and preventing its delamination. The oxidation of materials reduces the electrical conductivity of both the materials themselves and the composites based on them [34], which decreases the risk of galvanic corrosion.

Heteroatom doping can lead to either an increase in conductivity due to changes in the materials electronic structure or a decrease due to an increased number of defects in the graphene layers [35,36]. Structural defects and the curvature of graphene layers hinder the agglomeration of nanoflakes and improve mechanical interlocking with the polymer. Heteroatom doping alters the chemical reactivity of graphene, creating active sites [35] for stronger chemical or physical bonding with the PVC matrix. Theoretically, P-GNFs can act as active corrosion-inhibiting agents, since phosphorus is a well-known corrosion inhibitor [37]. Upon microscratch formation and subsequent moisture ingress, phosphorus-containing groups can be controllably released and form a protective passivating layer (e.g., iron phosphates) on the exposed metal surface, thereby imparting a self-healing capability to the coating [37].

Nitrogen doping of CNMs as fillers in anticorrosion coatings has been studied exemplified by graphene oxide and carbon nanotubes [6]. It is of great interest to investigate the effect of various methods of CNM modification (oxidation and doping) on the properties of anticorrosion coatings, focusing on water diffusion kinetics within the coating and novel methods for studying this process.

## 2. Materials and Methods

Few-layer graphene nanoflakes (GNF) and their nitrogen- and phosphorus-doped counterparts (N-GNFs and P-GNFs) were synthesized by pyrolysis of vapors of n-hexane (99%, Komponent-Reaktiv, Moscow, Russia), acetonitrile (99%, Komponent-Reaktiv, Moscow, Russia), and a 2% solution of triphenylphosphine (98%, Sisco Research Laboratories, Mumbai, India) in toluene (99.5%, Komponent-Reaktiv, Moscow, Russia) on an MgO template at 900 °C for 30 min [38,39]. Carbon nanotubes (CNT) were synthesized by pyrolysis of n-hexane vapors (99%, Komponent-Reaktiv, Moscow, Russia) on an MgO template in the presence of Co-Mo catalysts at 750 °C for 5 h [38]. The vapors were carried by a nitrogen flow (99.999%, NII KM, Moscow, Russia) into a quartz reactor of a Carbolite TZF 12/100/900 horizontal furnace (Carbolite, Hope, UK) by bubbling through a Drechsel flask containing the precursor at 1000 mL/min for GNF synthesis and 500 mL/min for CNT synthesis. Prior to synthesis, the reactor was purged with a nitrogen flow of 200 mL/min for 1 h to remove air. The obtained materials were washed from catalyst/template particles in a 15% hydrochloric acid solution under continuous stirring, rinsed with distilled water, and dried. CNTs and GNFs were refluxed in nitric acid for 3 h and 1.5 h, respectively, to obtain oxidized materials (CNTox and GNFox), washed free of acid, and dried. The morphology of the carbon material particles was investigated using a JEM-2100 transmission electron microscope (JEOL, Tokyo, Japan), and the chemical composition and surface atomic states were analyzed using an Axis Ultra DLD X-ray photoelectron spectrometer (Kratos Analytical, Manchester, UK), based on works [40,41,42]. The degree of material defectiveness was studied by Raman spectroscopy. The spectra were recorded using a LabRam HR800UV (Horiba Jobin Yvon, Longjumeau, France) spectrometer equipped with a confocal microscope. A He-Ne laser with an excitation wavelength of 632.81 nm was used. The background was subtracted from the spectra prior to calculating the intensity ratio of the D and G bands (*I*_D_/*I*_G_). The average distance between point defects, *L*_D_, and the areal defect density, *n*_D_, were calculated using the following relationships [43]:

(12) $$L_{D}^{2}\left(nm^{2}\right)=\left(1.8\pm 0.5\right)\cdot 10^{-9}\lambda_{L}^{2}{\left(\frac{I_{D}}{I_{G}}\right)}^{-1},$$

(13) $$n_{D}\left(cm^{-2}\right)=\frac{1.8\cdot 10^{22}}{\lambda_{L}^{4}}\left(\frac{I_{D}}{I_{G}}\right),$$

where λ_L_ is the laser excitation wavelength (in nm).

0.05 wt.% of carbon nanomaterials (N-GNFs, P-GNFs, GNFox, CNTox) were added to an antifouling enamel based on a vinyl chloride copolymer binder (a suspension of biocides in a solution of vinyl polymer modified with epoxy resin in organic solvents) XC-5226 (NTC Spetsemal LLC, St. Petersburg, Russia), and the resulting mixtures were ultrasonicated for 3.5 h. The original and modified enamels were applied to steel plates of grade PCA (GOST P 52927-2023, Novolipetsk Metallurgical Combine PJSC, Lipetsk, Russia) 4 mm thick, using a drawdown bar to control the coating thickness at about 100 μm. Prior to coating application, the plate surfaces were ground with sandpaper and wiped with alcohol. The applied enamel was dried at room temperature for 24 h. The coating layer thickness after drying was monitored with an RGK MC-25 micrometer (RGK, MoscowRussia) (resolution of 1 μm).

The process of water penetration into the anticorrosion coating upon immersion in a 3.5% sodium chloride solution simulating seawater was investigated. The amount of absorbed water was determined by diffuse reflectance infrared spectroscopy. Paint samples were applied to alloyed steel plates of 15×15 mm size. The plates were kept in the solution for specified times (from 1 to 9 days). Before measurement, the samples were removed from the solution, the surface was dried with strips of filter paper, and they were immediately placed in the spectrometer. Spectra were recorded on an EQUINOX 55/S FT-IR spectrometer (Bruker Optik GMBH, Ettlingen, Germany) with a Selector diffuse reflectance accessory (Specac, Orpington, Kent, UK). The obtained reflectance spectra were converted to Kubelka–Munk units and processed using OPUS 6.0 software (Bruker Optik GMBH, Ettlingen, Germany). Baseline correction was applied, and the spectra were normalized to the intensity of the absorption band at 2930 cm^−1^ corresponding to C-H stretching vibrations. For each paint, 4 independent measurements were performed, the intensity of the absorption peak around 3400 cm^−1^, corresponding to O-H stretching vibrations, was averaged, and the standard deviation of the mean was calculated.

The kinetics of water diffusion were also studied using a three-electrode cell filled with 3.5% sodium chloride solution and a VSP potentiostat (BioLogic, Seyssinet-Pariset, France). The working electrode (a steel plate with a coating and a window for contact with the solution) had a working surface area of 1 cm^2^. A silver–silver chloride reference electrode and a platinum auxiliary electrode with an area of 1 cm^2^ were used. For all coatings, the evolution of the open-circuit potential *E*_OCP_ over time was monitored by recording its values every 30 s, and for the paint with P-GNF filler, the open-circuit potential was recorded at 30 min intervals with 5 min pauses for EIS measurements (frequency range 20 mHz to 1 MHz, voltage amplitude 10 mV). For each time point, the Nyquist diagram was fitted using the model in Figure 1b, and the time dependences of the coating capacitance *C*_c_ and resistance *R*_c_, the double-layer capacitance *C*_dl_, and the charge-transfer resistance *R*_ct_ were calculated. The effective capacitance was calculated using formula (8), employing the values of the constant phase element parameters and resistance. The change in the degree of delamination α of the coating during the test was estimated from the breakpoint frequency *f*_b_ (*f*_b_ was calculated by definition) and the phase angle at 10 Hz θ_10Hz_, as well as the water diffusion rate *D* into the coating. To calculate the diffusion rate *D* the initial portion of the kinetic curve obtained from the Brasher–Kingsbury equation was fitted using Equation (4) in OriginPro 2021. The instantaneous standard deviation *SD*(*t*) of the high-frequency noise of the open-circuit potential* E*_OCP_ and the low-frequency trend line of the open-circuit potential $\bar{E_{OCP}\left(t\right)}$ were calculated from the upper and lower envelopes computed in OriginPro 2021:

(14) $$k\cdot SD\left(t\right)=\frac{Upper\;Envelope-Lower\;Envelope}{2}$$

(15) $$\bar{E_{OCP}\left(t\right)}=\frac{Upper\;Envelope+Lower\;Envelope}{2}$$

*k* is the crest factor (a coefficient depending on the noise distribution and the desired confidence level) [44]. For example, for Gaussian noise, *k* = 3 means that the envelope encompasses ~99.7% of all instantaneous noise values. Provided that the data are collected at a constant temperature and sampling rate, and the noise distribution is assumed to be stationary, the crest factor remains constant. A 5-point window was used for smoothing when constructing the envelopes to obtain relatively smooth curves while still capturing abrupt signal changes. The high-frequency open-circuit potential noise is

(16) $$OCP\;Noise\;\left(t\right)=E_{OCP}\left(t\right)-\bar{E_{OCP}\left(t\right)}$$

Prior to plotting the dependence $k\cdot SD\left(t\right)$, the data were additionally smoothed with a median percentile filter with a 200-point window in OriginPro 2021. Since the open-circuit potential noise is non-stationary, a short-time Fourier transform (STFT) with a Hanning window of 256 points length and 128 points overlap in OriginPro 2021 was used to represent it in the frequency domain and describe the evolution of amplitude over time. The amplitude was expressed in dB.

## 3. Results and Discussion

**Figure 2:**
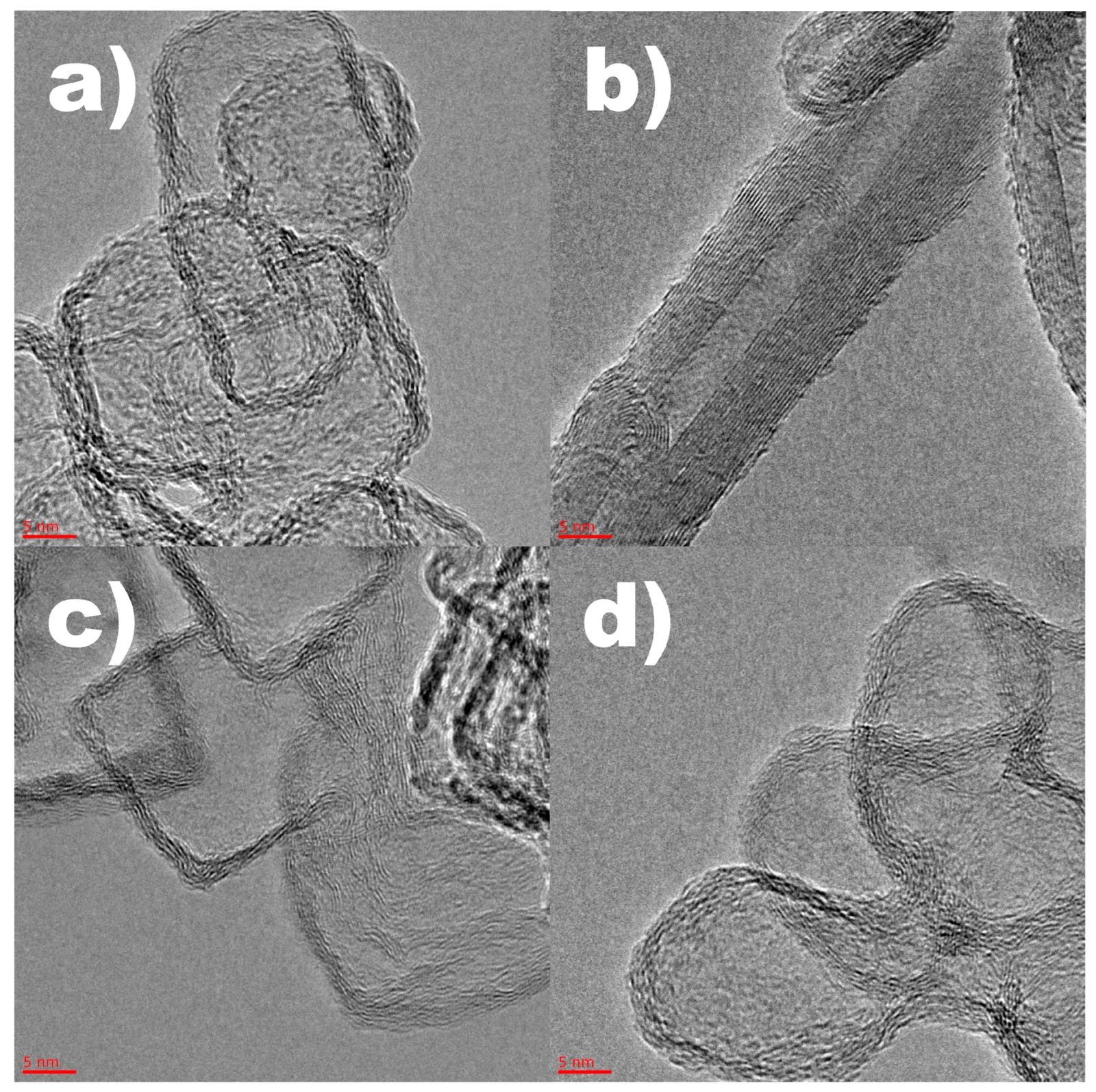
TEM images of (**a**) GNFox, (**b**) CNTox, (**c**) N-GNF, (**d**) P-GNF.

In our recent work [45], both the synthesized CNMs themselves and the anticorrosion properties of PVC-based paints with CNM fillers were comprehensively investigated; herein, we focus exclusively on the fundamental properties of the CNMs, illustrated by TEM images, X-ray photoelectron and Raman spectra. According to TEM data (Figure 2), GNFox, N-GNFs, and P-GNFs consist of rectangular stacks of graphene layers, approximately 20–40 nm in lateral size, with bent edges, while CNTox are multi-walled nanotubes with an outer diameter of approximately 15 nm and comprising 12–16 layers. Changes in material morphology and defect structure during the modification process have been investigated in detail in our laboratory’s previous studies [46,47,48]. The key point is that the oxidation of GNF occurs predominantly at the edges of graphene layers, and polar oxygen-containing functional groups cause the layers to repel each other and bend at the edges. The oxidation of CNMs is typically accompanied by the complete oxidation of amorphous carbon and fine particles. In the case of CNTs, this process is characterized by exfoliation of surface layers, cutting of long nanotubes, and opening of the tube ends. Nitrogen doping does not lead to a significant change in the planar graphene structure of N-GNF (Figure 2c), since the N-C bond length is close to the C-C bond length, minimizing lattice distortion. The planar structure of P-GNF (Figure 2d) is associated with the relatively low dopant content.

**Figure 3:**
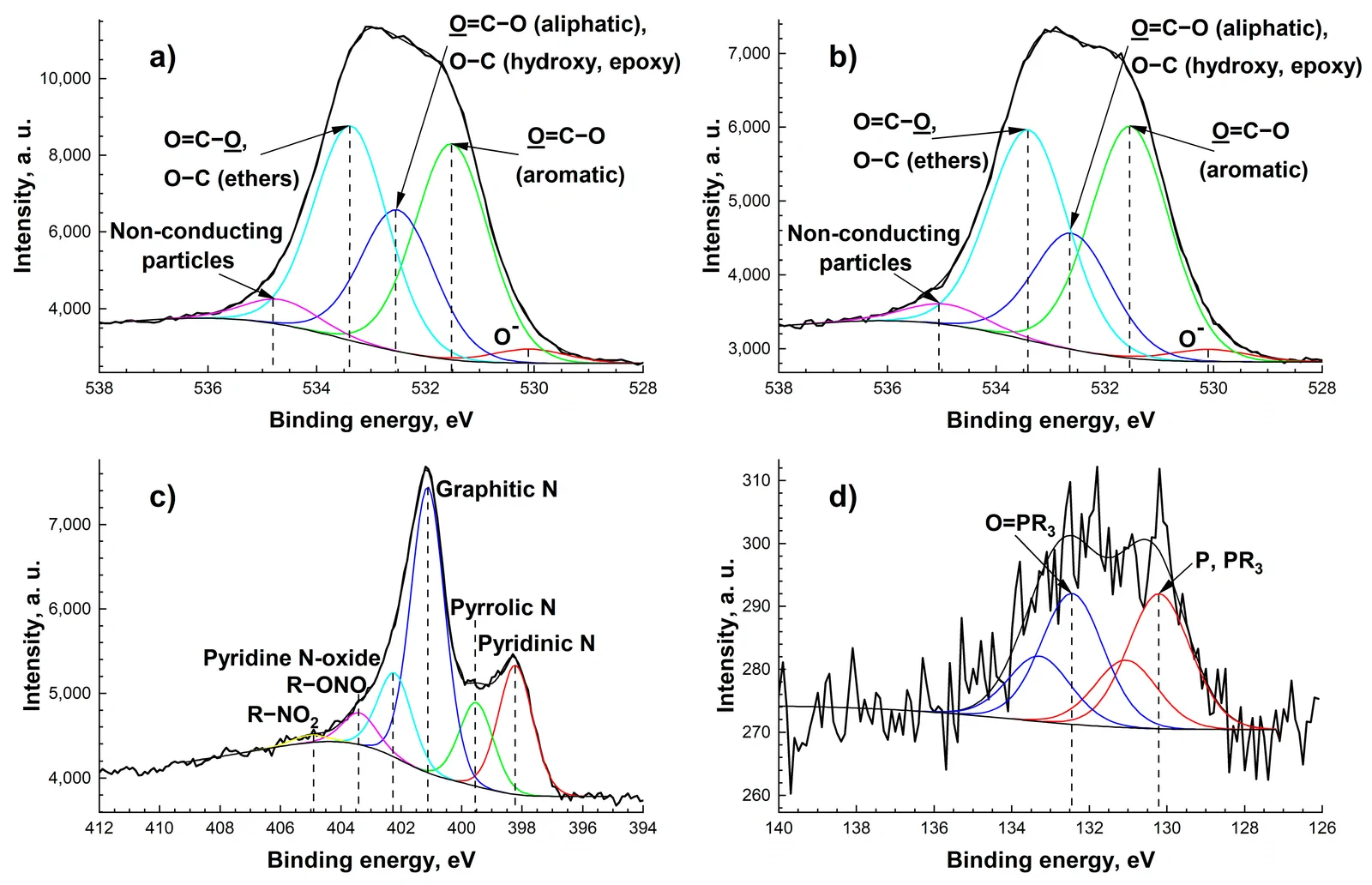
High-resolution XPS spectra: (**a**) O1s spectrum of GNFox, (**b**) O1s spectrum of CNTox, (**c**) N1s spectrum of N-GNF, (**d**) P2p spectrum of P-GNF.

According to XPS data [45], the carbon materials consist predominantly of sp^2^-hybridized carbon, characteristic of planar graphene structures. sp^3^-hybridized carbon is present in the structure of CNTox and GNFox, as it is inherent to the structure of CNTs with graphene sheets rolled into tubes, and surface oxidation is accompanied by an increase in sp^3^-hybridized carbon content. Oxygen on the surface of GNFox and CNTox (11.9 and 5.8 at. %, respectively) is predominantly in the form of carboxyl groups (Figure 3a,b). Nitrogen on the surface of N-GNFs (7.8 at. %) is present in the form of graphitic, pyridinic, pyrrolic, and, to a lesser extent, various oxidized nitrogen forms (Figure 3c). Phosphorus in P-GNFs is represented almost equally by incorporated phosphorus and its oxidized form (Figure 3d), although the phosphorus content is extremely low (<0.1 at. %).

**Figure 4:**
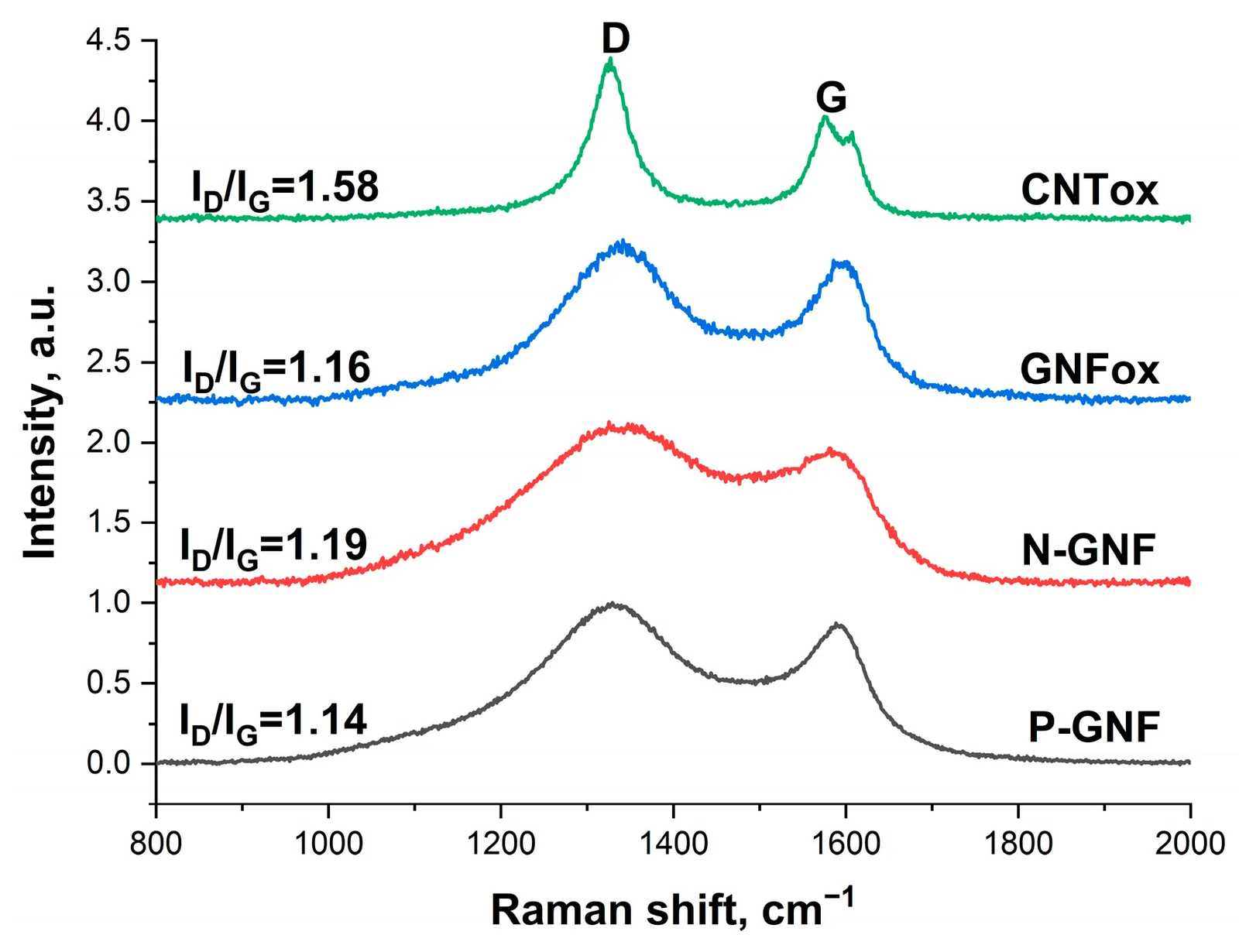
Raman spectra of CNTox, GNFox, N-GNF and P-GNF.

The defectiveness of modified CNMs was studied by Raman spectroscopy. In the Raman spectrum of CNTox, in addition to the narrow D (D1) and G bands at 1325 and 1575 cm^−1^, a peak is visible near 1605 cm^−1^ (Figure 4), corresponding to the D2 (D’) band [43,46,47]. The D2 (D’) band is often associated with the formation of small graphite or graphene clusters with a high edge-to-basal plane ratio [43,46,47]. Upon oxidation of CNTs, the intensity of the D2 (D’) band increases due to the partial exfoliation of the outer layers, while the D band intensification is attributed to the increased number of edge carbon atoms bonded to the functional groups [47]. In modified GNFs (GNFox, N-GNF, P-GNF), the G band is significantly shifted toward 1590–1600 cm^−1^, while the D band is broadened (Figure 4) due to an increased number of defects in graphene layers, a more disordered layer stacking compared to graphite or CNT [46] (according to XRD data, the graphite interlayer distance is smaller than in pristine and modified GNFs [46]) and the high particle surface-area-to-volume ratio. The shape of the N-GNF spectrum (Figure 4) indicates that D3 (D’’) and D4 bands located around 1450–1500 cm^−1^ and 1100–1200 cm^−1^ are very pronounced. This indicates a high content of amorphous carbon in the material. The significant difference in the *I*_D_/*I*_G_ ratio between CNTox and modified GNFs (Figure 4) is due to the difference in the particle geometry of the materials. The cylindrical graphene layers structure of CNTs contributes to an increase in the intensity of D band. The average distance between point defects *L*_D_ is 16 nm for modified GNFs and 14 nm for CNTox, which is comparable to the lateral dimensions of the material particles, because the defects are located predominantly at the particle edges. The average defect density *n*_D_ is 1.3·10^11^ cm^−2^ for modified GNFs and 1.8·10^11^ cm^−2^ for CNTox.

**Figure 5:**
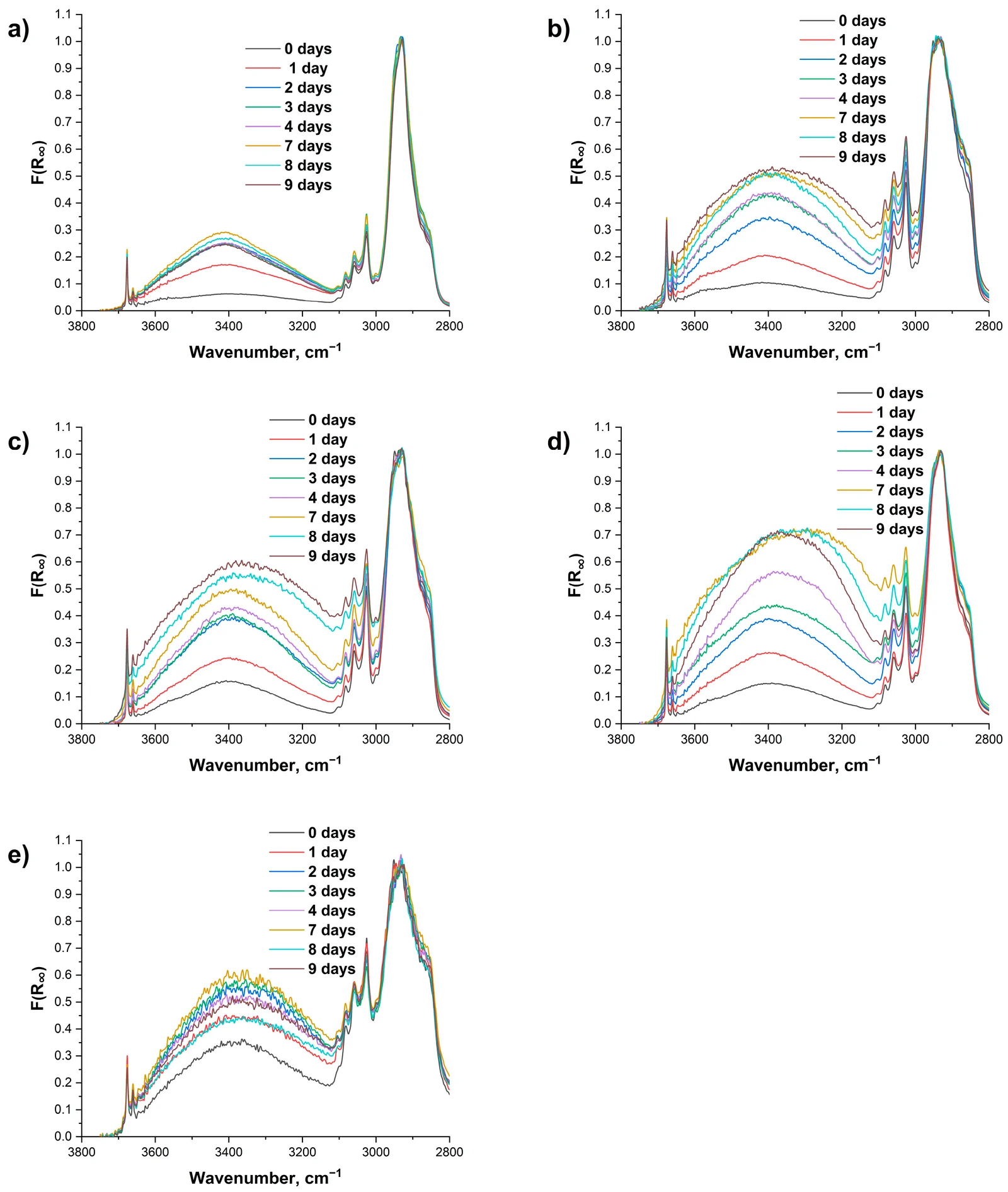
Diffuse reflectance spectra *F*(*R*_∞_) normalized to the intensity of the 2930 cm^−1^ absorption band for coatings with fillers of (**a**) GNFox, (**b**) CNTox, (**c**) N-GNFs, (**d**) P-GNFs, and (**e**) the pristine enamel, kept in salt solution for 0 to 9 days.

**Figure 6:**
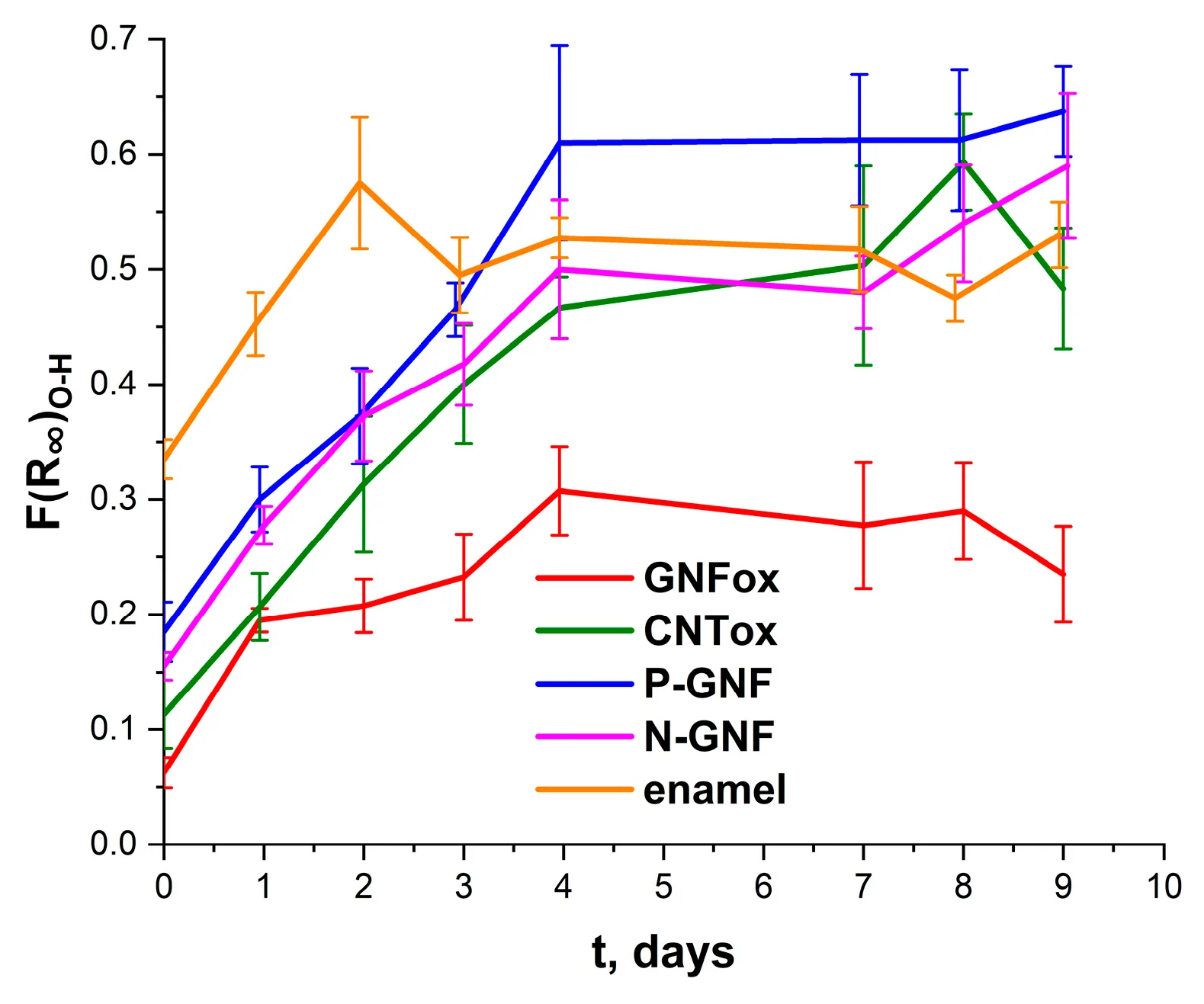
Intensity of the 3400 cm^−1^ absorption band in diffuse reflectance spectra *F*(*R*_∞_)_O-H_ for coatings with fillers of GNFox, CNTox, N-GNFs, P-GNFs, and the pristine enamel, kept in salt solution for 0 to 9 days (average of 4 independent measurements).

The kinetics of diffusion of 3.5% sodium chloride solution into the original coating and coatings with carbon nanomaterial additives were monitored using diffuse reflectance infrared spectroscopy. The diffuse reflectance spectra, expressed in Kubelka–Munk units *F*(*R*_∞_), were normalized to the intensity of the absorption band at 2930 cm^−1^, corresponding to the C-H stretching vibrations of the enamel (Figure 5). With this normalization, the intensity of the absorption band around 3400 cm^−1^, corresponding to O-H stretching vibrations, equals the ratio of the intensities of the O-H and C-H bands, i.e., it correlates with the water content in the paint. It is assumed that the intensity of the C-H band does not change upon interaction with water. The outstanding barrier characteristics of GNFox can be seen from a comparison of the dynamics of the absorption peak intensity around *F*(*R*_∞_)_O-H_ for the original enamel and those with carbon material fillers (Figure 5): the enamel with GNFox has a substantially lower maximum water capacity in the series of samples, and the equilibrium capacity is reached on average after 4 days, compared to 2 days for the original paint and 4–9 days for the paint with CNM additives. Additions of oxidized materials (GNFox and CNTox) reduce the maximum water capacity, whereas additions of doped materials (N-GNF and P-GNF) increase it (Figure 5 and Figure 6). The difference in the available pore volume in the enamel is due to the difference in the total pore volume of the CNMs (measured previously by nitrogen adsorption) used as fillers [45].

Qualitatively, the greater the difference between the IR spectra at different immersion times (Figure 5), the slower the water diffusion into the paint, and the better the barrier properties. Thus, CNM additives reduce the water diffusion rate into the coating, and the water diffusion rate into the enamel with oxidized materials is lower than with doped materials, since the diffusion rate is determined by the available pore volume. Although the reflectance IR spectroscopy data contain only 8 points corresponding to different immersion times of the coating in 3.5% NaCl solution, they can be used to estimate the water diffusion rate *D*. For Fickian diffusion, the following holds [16]:

(17) $$D=\frac{d^{2}}{t_{S}},$$

where *t*_S_ is the time required for saturation of water uptake. With a coating thickness of *d* = 50 μm, the estimate yields *D* = 1.4·10^−10^ cm^2^/c for the pristine enamel and (3–7)·10^−11^ cm^2^/c for enamels with CNM additives, which falls within the range of 10^−8^–10^−12^ cm^2^/c for coatings with good barrier properties at room temperature reported in the literature [16].

**Figure 7:**
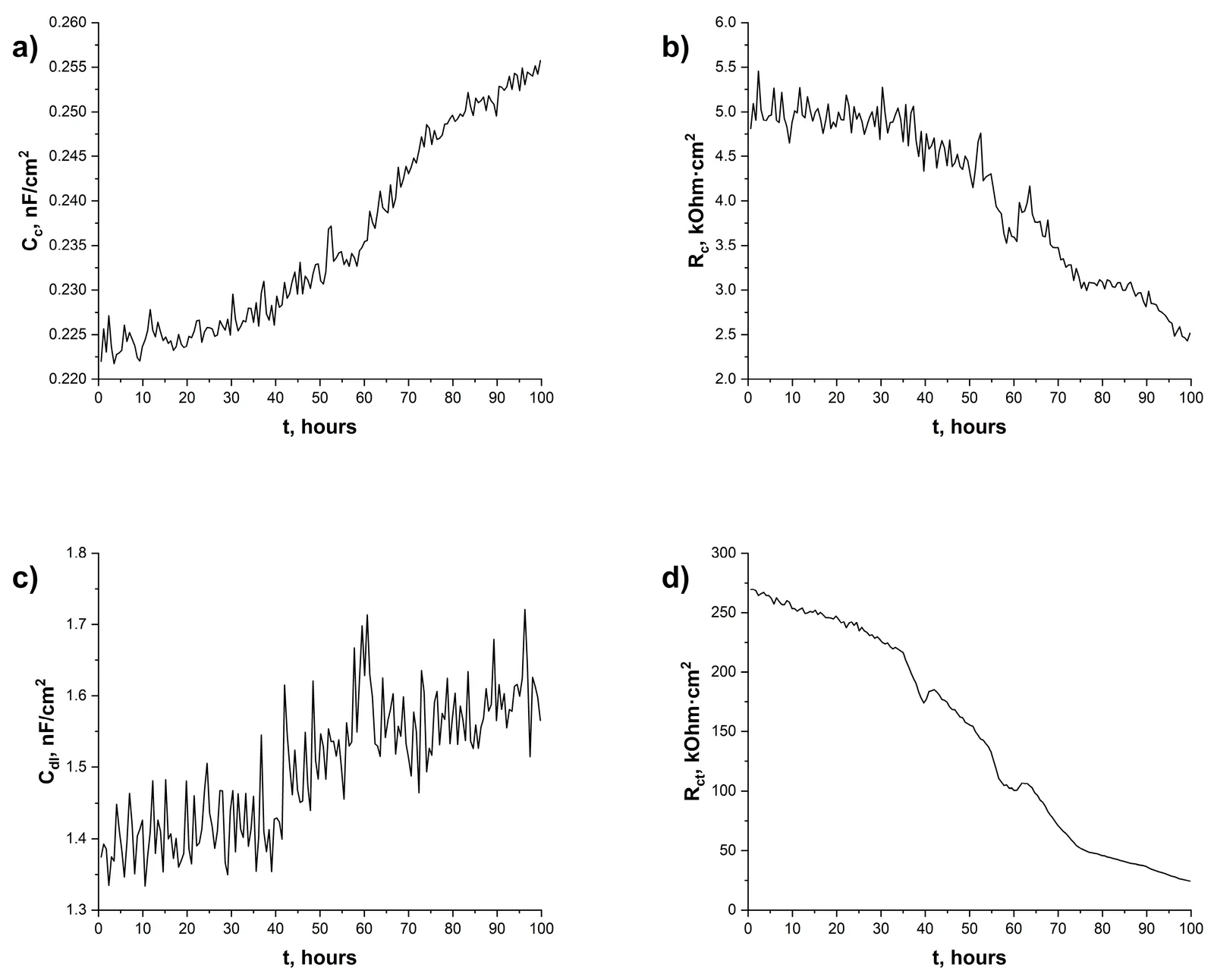
Time dependence of (**a**) *C*_c_, (**b**) *R*_c_, (**c**) *C*_dl_ and (**d**) *R*_ct_ for a steel plate coated with enamel with P-GNF upon immersion in 3.5% NaCl solution.

**Figure 8:**
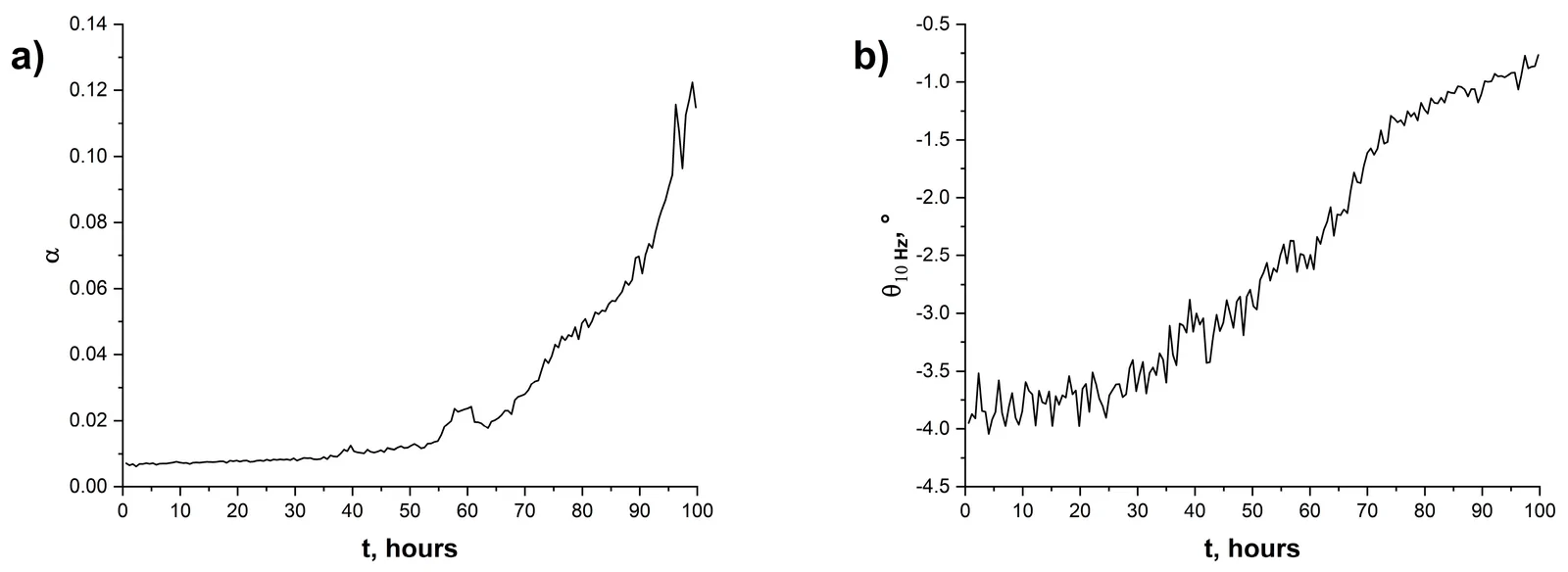
Time dependence of (**a**) the degree of delamination α calculated from the breakpoint frequency *f*_b_, and (**b**) the phase angle at 10 Hz θ_10Hz_ for a steel plate coated with enamel with P-GNF upon immersion in 3.5% NaCl solution.

The evolution of the equivalent circuit parameters describing the behavior of the enamel coating with 0.05% P-GNF additive in 3.5% NaCl solution as water diffuses into the coating is shown in Figure 7. The coating capacitance *C*_c_ increases due to the increasing water content in the enamel, reaching 3.5% by volume (from the Brasher–Kingsbury equation in Equation (2)) after 100 h of immersion in the solution. The coating resistance *R*_c_ correspondingly decreases. Since the coating constant phase element behaves as an ideal capacitance with *n* = 1, for calculating the effective double-layer capacitance *C*_dl_ using formula (8), due to its non-ideality, it was necessary to take into account *Y*_dl_, the exponent *n*_dl_ and the charge-transfer resistance *R*_ct_. The double-layer capacitance *C*_dl_ remains constant within the noise level and increases sharply after 40 and 60 h of immersion in the solution. At the same time, against the background of the general trend of monotonic decrease in charge-transfer resistance *R*_ct,_ minima are observed around 40 and 60 h. To understand the reasons for this non-monotonicity, it is necessary to construct the dependence of the degree of delamination α on time. The degree of delamination α, calculated from the breakpoint frequency *f*_b_, increases with immersion time (Figure 8a), but the general trend curve shows two maxima around 40 and 60 h, associated with the dynamic nature of the delamination process and corrosion of the metal under the coating (under the action of osmotic pressure at the metal surface–protective coating interface, blisters form and collapse [49,50], a phenomenon called blistering). The phase angle at 10 Hz θ_10Hz_ (Figure 8b), which is also associated with delamination, increases with immersion time, but against the background of noise, the two maxima are barely visible. The change in the degree of coating delamination α = *A*_d_/*A* leads to corresponding changes in *R*_c_, *R*_ct_, and *C*_dl_ in accordance with the simplified expressions for the equivalent electrical circuit parameters from [24]:

(18) $$C_{c}=\frac{\varepsilon \varepsilon_{0}A}{d}$$

(19) $$R_{c}=\frac{\rho d}{A_{d}}$$

(20) $$R_{ct}=\frac{R_{ct}^{0}}{A_{d}}$$

(21) $$C_{dl}=C_{dl}^{0}A_{d}$$

With increasing α, *R*_ct_ decreases and *C*_dl_ increases. An increase in the degree of delamination also leads to a decrease in *R*_c_, but the decrease in coating resistance is also affected by a decrease in electrical resistivity ρ due to water diffusion and possible film swelling (change in coating thickness *d*).

**Figure 9:**
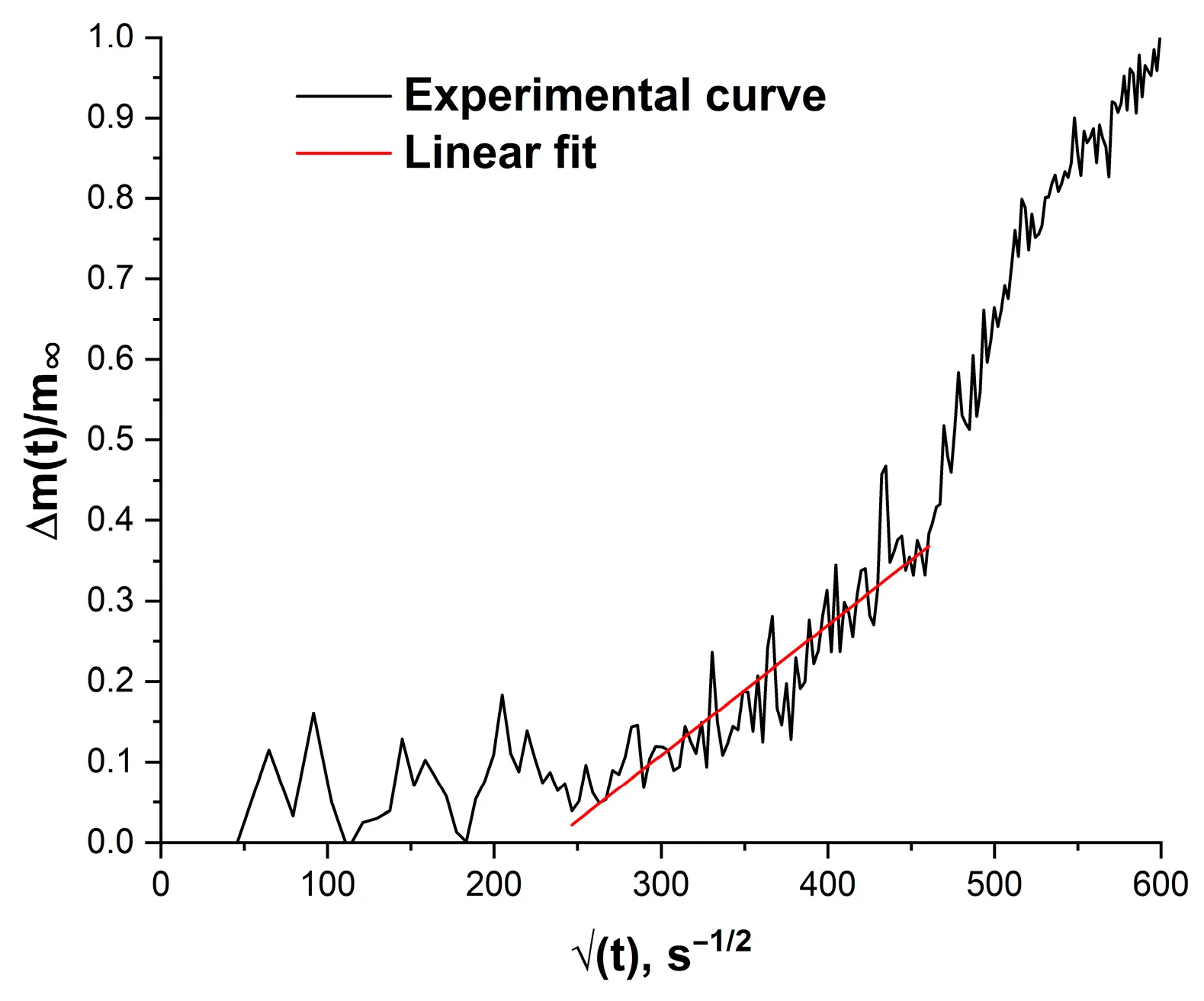
Kinetic curve calculated using the Brasher–Kingsbury equation for the enamel coating with P-GNF additive and the linear approximation of the initial segment.

The high values of the degree of delamination (from 0.007 to 0.12) and the low values of the low-frequency impedance (2.8·10^5^ Ohm·cm^2^ and below) indicate substantial degradation of the protective coating by the start of recording the time evolution of the impedance spectra. This is because the enamel coating with 0.05% P-GNF additive was kept in 3.5% NaCl solution for 12.6 days before impedance measurements of the initially high-resistance coating, exceeding the input impedance of the potentiostat, became possible (Figure S2). The kinetic curve (Figure 9), calculated using the Brasher–Kingsbury equation from the time dependence of the coating capacitance *C*_c_, shows an induction period of about 17 h preceding the linear initial stage of mass increase. In total, about 13 days precede the water diffusion observed from the change in coating capacitance. The water diffusion rate into the coating of thickness *d* = 50 μm, calculated from the linearized initial segment of the kinetic curve (Figure 9), is *D* = (5.2 ± 1.2)·10^−11^ cm^2^/s. The estimate obtained from the diffuse reflectance IR spectroscopy data (7·10^−11^ cm^2^/s) is of the same order of magnitude.

**Figure 10:**
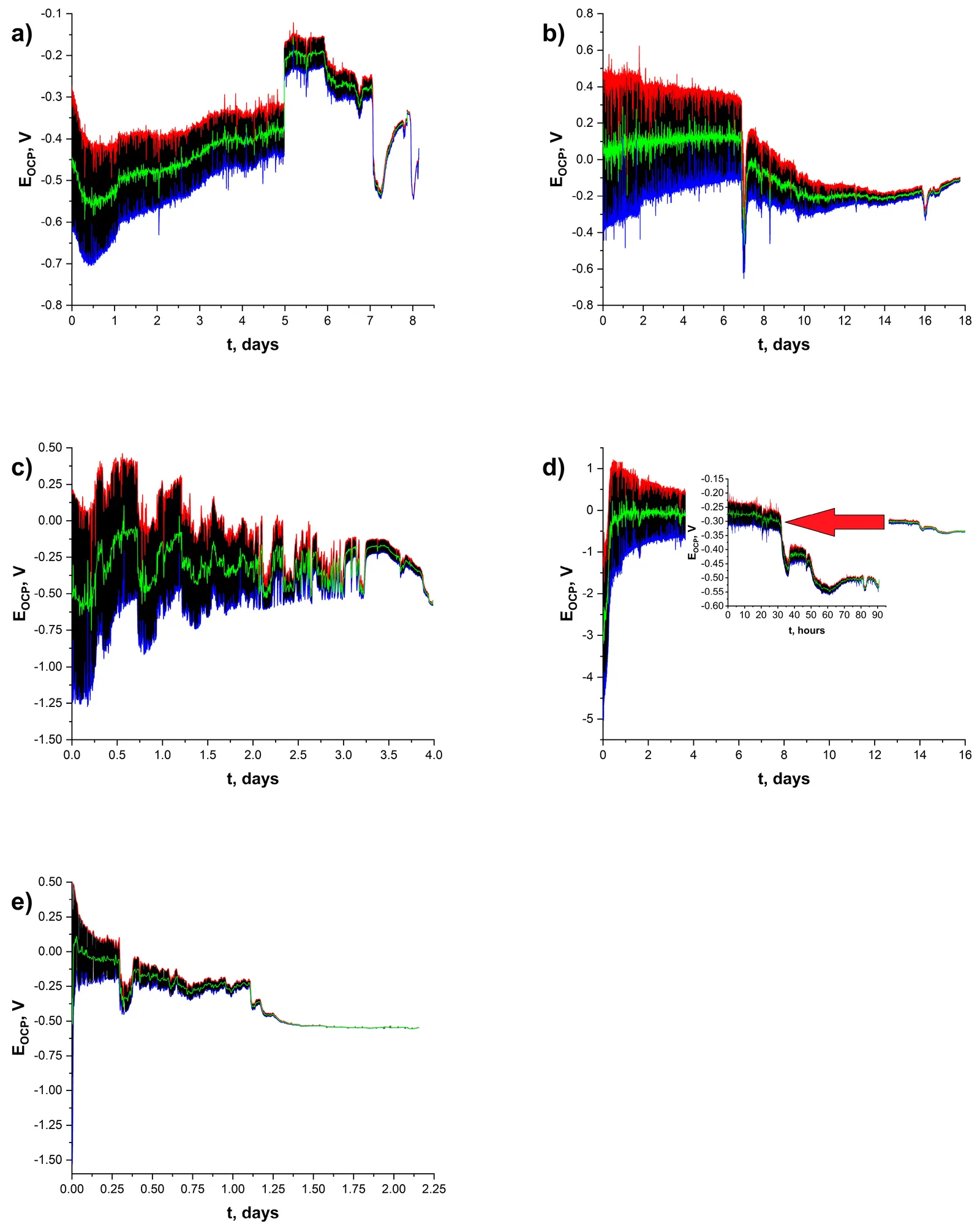
Open-circuit potential *E*_OCP_ for a cell with an anticorrosion coating (XC-5226 enamel with 0.05 wt.% additives of (**a**) GNFox, (**b**) CNTox, (**c**) N-GNF, (**d**) P-GNF, and (**e**) the pristine enamel without additives) as a function of immersion time *t* in 3.5% NaCl solution. Black denotes the experimental curve, red the upper envelope, blue the lower envelope, and green the open-circuit potential trend line $\bar{E_{OCP}\left(t\right)}$.

In contrast to EIS, the evolution of the OCP of a three-electrode cell can be measured at the earliest stages of water uptake by the coating and its degradation. The open-circuit potential *E*_OCP_ (Figure 10) is significantly affected by noise, but it is precisely the noise that carries useful information about the state of the coating and is attracting increasing attention [50,51,52,53,54,55]. To extract the high-frequency noise from the signal, the trend line [53,54] of the open-circuit potential $\bar{E_{OCP}\left(t\right)}$ should be calculated. Using formula (15) from the upper and lower envelopes (shown in red and blue in Figure 10) of the signal (shown in black), the calculation was performed (shown in green). The change in $\bar{E_{OCP}\left(t\right)}$ is due to a set of physicochemical changes at the metal–coating–electrolyte interface [40]. In addition to water diffusion through micropores and coating defects, ions (primarily aggressive chloride ions Cl^−^ from the salt solution) also penetrate to the metal surface. As soon as water and ions reach the metal–coating interface, electrochemical corrosion begins. When the electrolyte contacts the unprotected metal, anodic sites are activated, where metal dissolution begins (Fe → Fe^2+^ + 2e^−^). This causes $\bar{E_{OCP}\left(t\right)}$ to shift sharply toward more negative values. In other areas under the film, the cathodic reaction proceeds (typically the reduction of dissolved oxygen: O_2_ + 2H_2_O + 4e^−^ → 4OH^−^). Local pH changes (alkalinization at cathodic sites) can lead to the disruption of adhesive bonds between the polymer and the metal and to delamination. Exposure of new metal areas also causes potential jumps. The formation of rust or other corrosion products under the film changes the local chemical environment. Corrosion products can create additional diffusion resistance (screen the metal), which sometimes leads to temporary stabilization or a slight positive shift in potential. However, more often they cause osmotic pressure, leading to blister formation, which can cause mechanical rupture of the coating, again exposing the metal and causing the potential to drop to negative values. In general, step changes in the open-circuit potential are often associated with the formation of new pores, microcracks, and local delamination of the coating. Figure 10d shows an enlarged fragment of the *E*_OCP_(*t*) dependence corresponding to the time of EIS measurements (start after 12.6 days of immersion) of the enamel with P-GNF additive. It can be seen that the maxima in the degree of delamination around 40 and 60 h of immersion (Figure 8a) are preceded by a sharp decrease in $\bar{E_{OCP}\left(t\right)}$.

**Figure 11:**
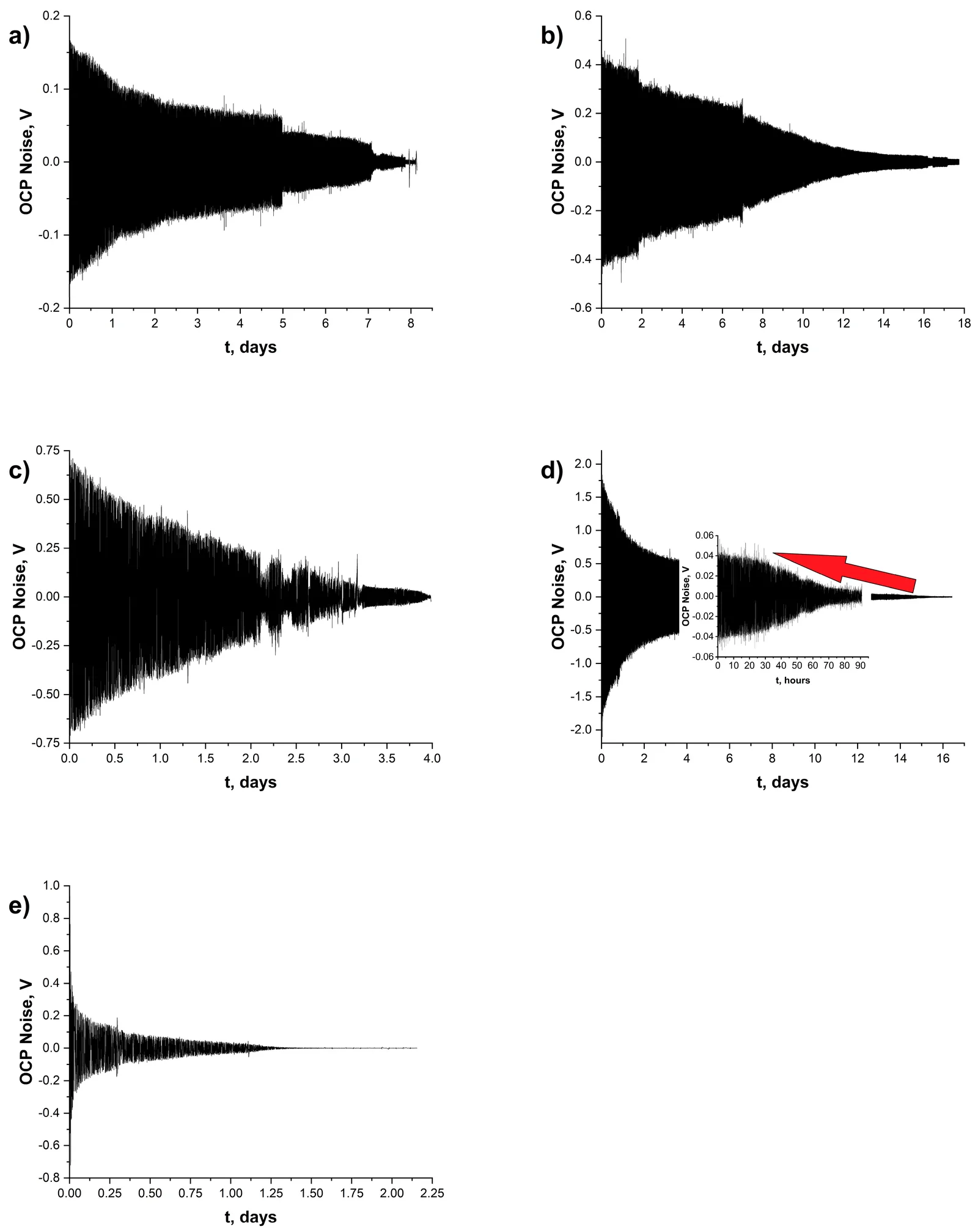
OCP noise obtained by subtracting the trend line from the open-circuit potential for a cell with an anticorrosion coating (XC-5226 enamel with 0.05 wt.% additives of (**a**) GNFox, (**b**) CNTox, (**c**) N-GNF, (**d**) P-GNF, and (**e**) the pristine enamel without additives) as a function of immersion time *t* in 3.5% NaCl solution.

**Figure 12:**
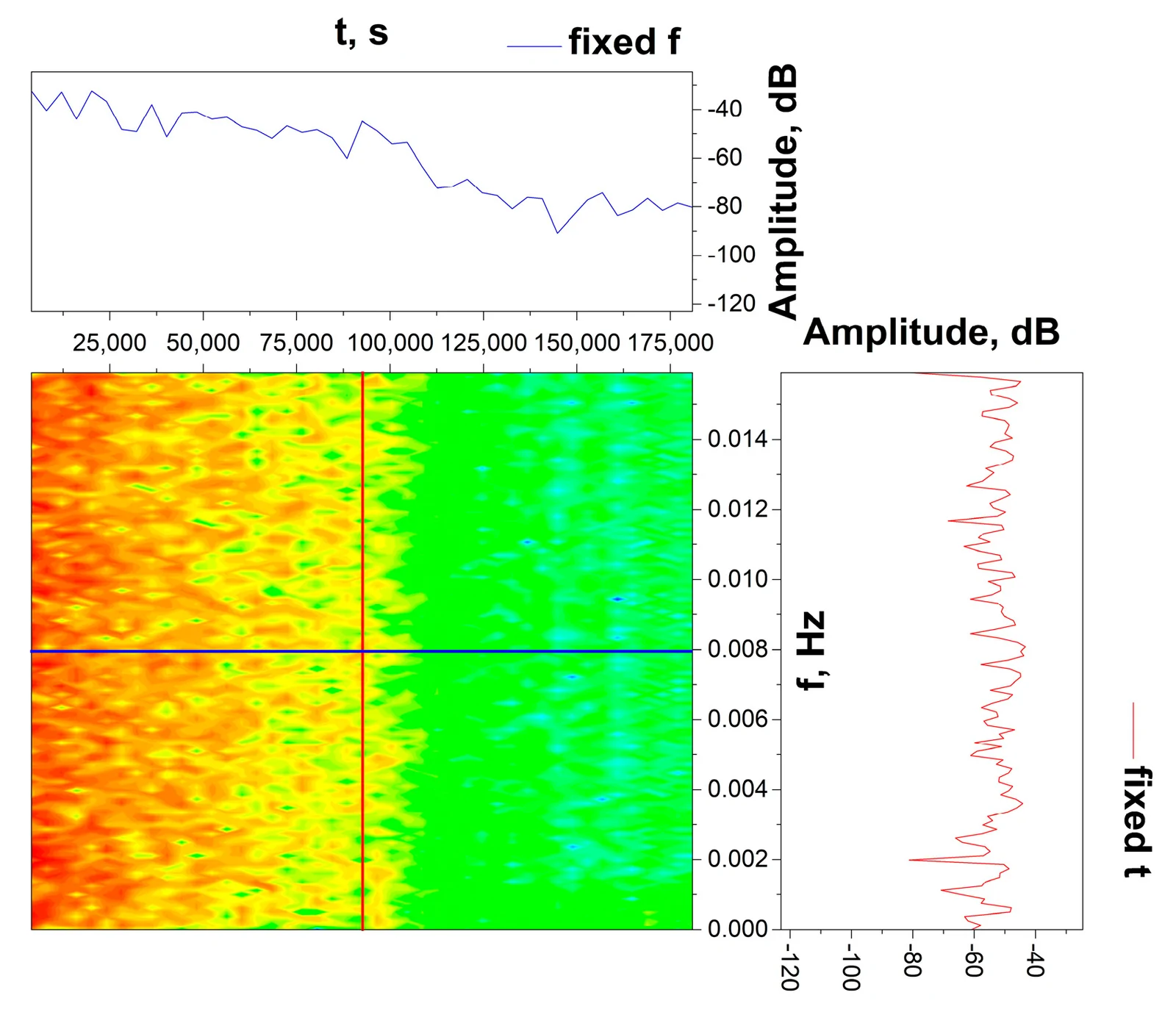
Result of the short-time Fourier transform for the OCP noise of the pristine enamel coating and its cross-sections at a fixed noise frequency and at a fixed time.

**Figure 13:**
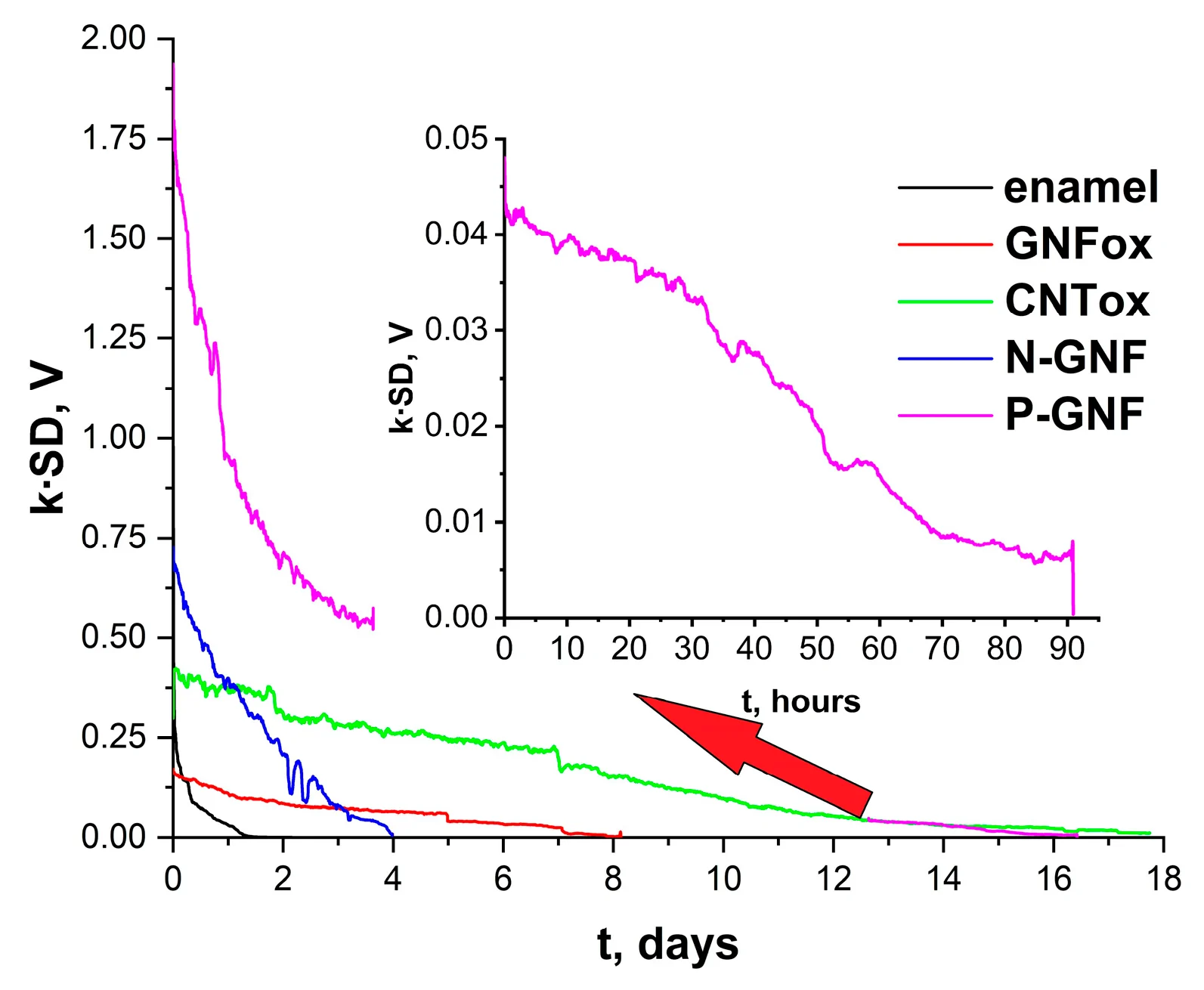
Product of the crest factor and the standard deviation of the OCP noise *k*·*SD* for a cell with an anticorrosion coating (XC-5226 enamel and the same with 0.05 wt.% additives of GNFox, CNTox, N-GNF, P-GNF) as a function of immersion time *t* in 3.5% NaCl solution.

The OCP noise, obtained by subtracting the trend line (formula (16)), is shown in Figure 11. As the immersion time of the coatings in the salt solution increases, the noise amplitude decreases, and the noise spectrum at any time is white noise (Figure 12). The standard deviation of the noise *SD*, calculated using formula (14), decreases as the immersion time of the coatings in the salt solution increases (Figure 13). An enlarged fragment of the dependence of the standard deviation of the OCP noise for the paint with P-GNF additive (Figure 13), corresponding to the time of EIS measurements (start after 12.6 days of immersion), resembles the profile of *R*_ct_ in shape (Figure 7d). It can be assumed that the main contribution to the noise is thermal noise, since its spectrum is white noise, and the root-mean-square deviation of the potential noise is determined by the value of the active resistance [56]:

(22) $$SD=\;\sqrt{4k_{B}TR\Delta f},$$

where *k*_B_ is the Boltzmann constant, *T* is the temperature, *R* is the active resistance of the circuit, and Δ*f* is the frequency bandwidth in which the potential noise is recorded. In our case:

(23) $$R=\;R_{s}+R_{c}+R_{ct}\approx R_{c}+R_{ct},$$

due to the salt solution resistance *R*_s_ is much less than the coating resistance *R*_c_ and the charge-transfer resistance *R*_ct_. According to the EIS data (Figure 7b,d), the main contribution to the active resistance comes from the charge-transfer resistance *R*_ct_. According to Equation (20), the charge-transfer resistance *R*_ct_. is inversely proportional to the degree of delamination α. Consequently, the observed reduction in noise amplitude is directly attributable to the progression of delamination. This explains the behavior of the enlarged fragment of the *k*·*SD* dependence (start after 12.6 days of immersion): the formation and collapse of blisters in the coating, accompanied by two local minima against the background of the general decreasing trend of the standard deviation.

At the early stage of water diffusion into the coating, when saturation has not yet been reached, corrosion is low [7], and it can be assumed that at this stage the contribution of electrochemical noise (caused by spontaneous fluctuations in the rate of the electrochemical reactions at phase boundary due to the destruction and restoration of the protective oxide film, the formation of microscopic ulcers (pitting), the release of gas bubbles, and the adsorption of ions [50,51,52,53,54,55]) to the OCP noise is small. It consists of low-frequency irregular noise of large amplitude against the background of high-frequency thermal noise (caused by chaotic thermal motion of charge carriers [56]), which sets the main trend of the OCP noise. To verify how well subtracting the trend line from the OCP dependence isolates the thermal noise from the total noise, it is proposed to calculate (*k*·*SD*)^2^/*R*, which, according to relationships (22) and (23), equals:

(24) $$\frac{{\left(k\cdot SD\right)}^{2}}{R}\approx \frac{{\left(k\cdot SD\right)}^{2}}{R_{c}+R_{ct}}=const$$

**Figure 14:**
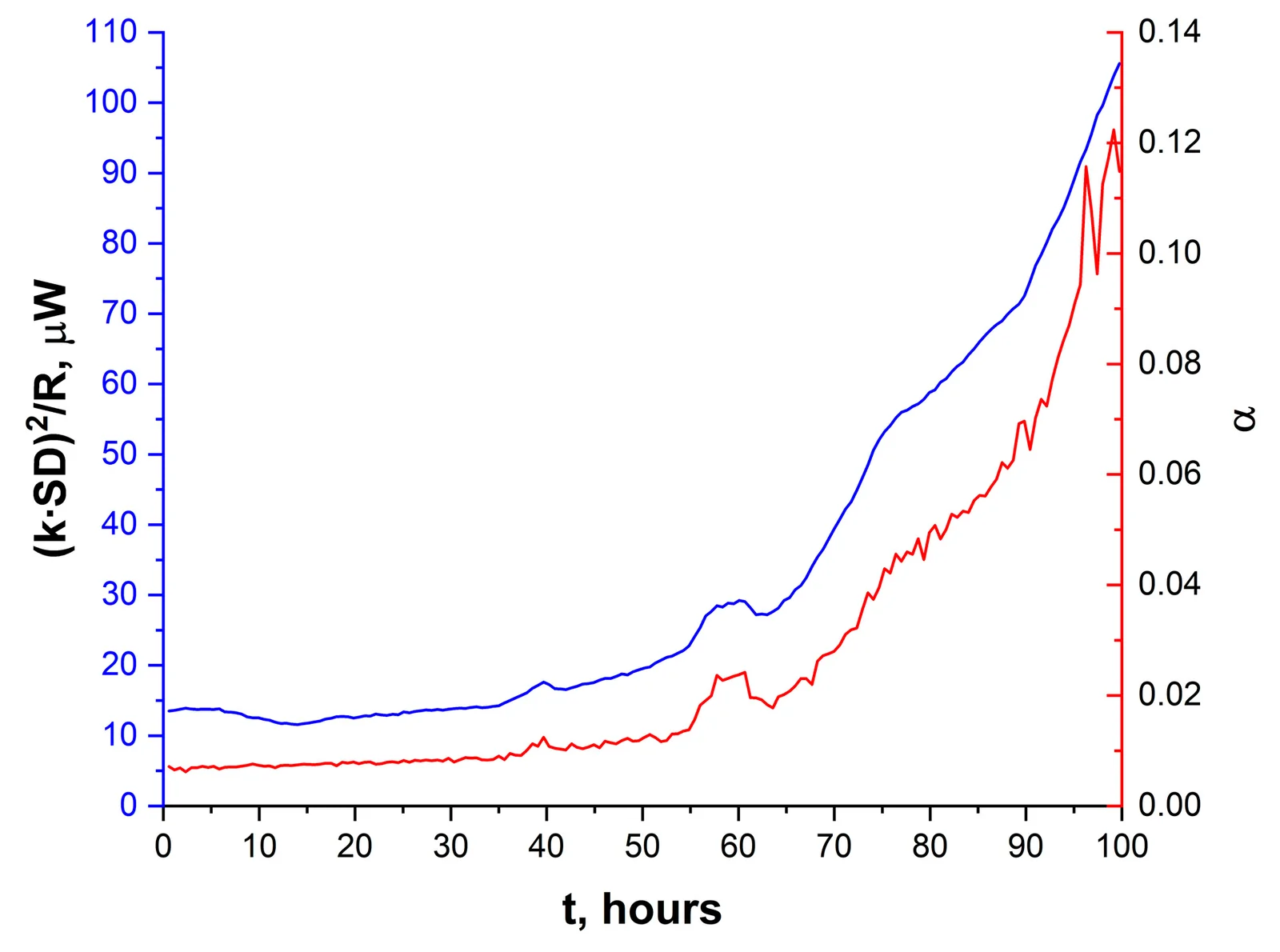
Comparison of the time dependence of the ratio (*k*·*SD*)^2^/*R* and the degree of delamination α for the XC-5226 enamel coating with 0.05 wt.% P-GNF additive upon immersion in 3.5% NaCl solution after 12.6 days of immersion.

For purely thermal noise, this value has the physical meaning of thermal noise power. Calculations for the interval following 12.6 days of immersion (Figure 14) reveal that during the first 35 h, when the degree of delamination α is small and remains relatively constant (according to Equation (10), *R*_c_*C*_c_ ≈ const), thermal noise predominates ((*k*·*SD*)^2^/*R = const*). Consequently, throughout the 14-day period following immersion, the noise amplitude is governed by Equation (22) and is proportional to $\sqrt{R}$. However, as soon as the first blister forms, with increasing delamination, the frequency and amplitude of electrochemical noise caused by steel corrosion increase. Subsequently, delamination and electrochemical noise with it only grow. Nevertheless, from the ratio (*k*·*SD*)^2^ at the initial moment of immersion in the salt solution (the start of OCP recording) and at the moment of the start of EIS measurements, and from the active resistance at the start of impedance measurements, the initial resistance *R*(0) of the circuit (essentially, of the coating itself, since the electrical double layer has not yet formed) for the coating of XC-5226 with 0.05 wt.% P-GNF additive can be estimated. Similarly, knowing *R*(0) of the enamel coating with P-GNF additive and the ratios (*k*·*SD*)^2^ in the series of coatings, *R*(0) for the other coatings can be calculated. The calculation result is given in Table 1. The initial resistance of the coating *R*(0) of the enamel with P-GNF additive reaches 640 MOm·cm^2^, so it is not surprising that it was impossible to record impedance from the very beginning of immersion in the salt solution, since the input resistance of the instrument is lower. However, a high active resistance of the circuit by itself does not guarantee the quality of the coating. For example, the original enamel has a high resistance but degrades in just 2 days (Table 1, Figure 13). The best ratio of initial coating resistance *R*(0) to its degradation time τ_∞_ (average rate of resistance degradation) is found in paints with oxidized CNMs, especially GNFox (Table 1). This correlates with the results of a previous work [45] devoted to these compositions. Thus, the coating efficiency *P* decreases in the order GNFox > CNTox > enamel > P-GNF > N-GNF. Additions of oxidized materials increase the polarization resistance *R*_p_ compared to the original enamel, whereas doped materials decrease it. At the same time, the corrosion rate of enamels with oxidized material additives, especially GNFox, is minimal, and that with N-GNFs is maximal. The formation of large blisters containing corrosion products within the N-GNF-modified coating is corroborated by laser scanning confocal microscopy (LSCM) data [45] (Figure S5) and the appearance of two sharp minima on the *k*·*SD*(*t*) curve after two days of immersion (Figure 13). The exceptional barrier properties of GNFox are attributed to the presence of numerous oxygen-containing functional groups, which ensure superior dispersion within the enamel matrix and facilitate hydrogen bonding with the polymer chains [33].

Unfortunately, for the active resistance of the cell, there is no simple and convenient expression such as the Brasher–Kingsbury equation for the coating capacitance to calculate the water diffusion rate *D* into the coating. The water content in the coating could be attempted to be accounted for using Archie’s law [57,58] or the tortuosity pore model [59], but the task is extremely complex, and accounting for the charge-transfer resistance *R*_ct_ within these models is not possible. Nevertheless, the benefit of observing the dynamics of the active resistance of the three-electrode cell and the degree of delamination using the open-circuit potential noise is evident.

## 4. Conclusions

The feasibility of studying the kinetics of water diffusion into an anticorrosion coating using diffuse reflectance infrared spectroscopy has been demonstrated. The estimated water diffusion rate into the coating is *D* = 1.4·10^−10^ cm^2^/s for the original enamel and (3–7)·10^−11^ cm^2^/s for enamels with CNM additives, which falls within the range of 10^−8^–10^−12^ cm^2^/s for coatings with good barrier properties at room temperature reported in the literature. Additions of oxidized materials (GNFox and CNTox) reduce the maximum water capacity, whereas additions of doped materials (N-GNF and P-GNF) increase it. The difference in the available pore volume in the enamel is due to the difference in the total pore volume of the CNMs. CNM additives reduce the water diffusion rate into the coating, and the water diffusion rate into the enamel with oxidized materials is lower than with doped materials, since the diffusion rate is determined by the available pore volume.

An algorithm for extracting the trend line and noise of the open-circuit potential of a three-electrode cell has been proposed. Changes in the trend line and the standard deviation of the noise provide information about coating delamination, and at low delamination, the change in the standard deviation of the noise can be used to calculate the relative change in the active resistance of the circuit *R*, approximately equal to the sum of the coating resistance *R*_c_ and the charge-transfer resistance *R*_ct_. If the active resistance of one of the coatings at low delamination is known from EIS, the absolute value of the active resistance of all coatings in the series can be calculated, provided that the data are collected and processed uniformly (which ensures equality of the crest factors for different *E*_OCP_ curves). Thus, the calculation yielded a high coating resistance at the initial time (*R*(0) = 640 MOhm·cm^2^) for the enamel with 0.5% P-GNF additive; however, it was shown that when comparing coating qualities, the time to complete coating degradation should also be compared. In terms of the ratio of the active resistance of the coating to the time to complete degradation, enamels with oxidized material additives (GNFox, CNTox) stand out favorably. Diffuse reflectance IR spectroscopy and OCP noise analysis demonstrated that the coating containing 0.5 wt% GNFox exhibits superior barrier properties, specifically characterized by low moisture absorption, a reduced water diffusion rate, high coating resistance, and a slow degradation rate. These enhanced properties are attributed to the high density of oxygen-containing functional groups, which facilitate superior particle dispersion within the polymer matrix and promote strong interfacial adhesion to the polymer chains via hydrogen bonding.

## Figures and Tables

**Table 1:** Initial active resistance *R*(0) of the three-electrode cell with a working electrode in the form of an anticorrosion coating of the pristine enamel and with CNM additives, immersed in 3.5% NaCl solution, time of active resistance degradation τ_∞_ (time for *k*·*SD* to decrease to zero) and their ratio *R*(0)/τ_∞_.

	P-GNF	N-GNF	Enamel	GNFox	CNTox
***R*** **(0), MOhm·cm^2^**	640	90	170	5	30
**τ_∞_, days**	16	4	2	8	18
***R*** **(0)/τ_∞_, Ohm·cm^2^/s**	460	260	1000	7	19

## Data Availability

The raw data supporting the conclusions of this article will be made available by the authors upon request.

## Supplementary Materials

The following supporting information can be downloaded at https://www.mdpi.com/article/10.3390/nano16211351/s1, which contains Figure S1 (Characteristics of the enamel XC-5226), Figure S2 (Nyquist plots of the steel plate coated with the P-GNF-modified enamel upon immersion in 3.5% NaCl solution) and Figures S3–S5 (Laser scanning confocal microscopy (LSCM) images of the coatings before and after electrochemical tests in 3.5% NaCl solution).

## Abbreviations

The following abbreviations are used in this manuscript:

CNM	Carbon nanomaterials
CNT	Carbon nanotubes
EIS	Electrochemical impedance spectroscopy
GNF	Graphene nanoflakes
IR	Infrared
OCP	Open-circuit potential
TEM	Transmission electron microscopy
XPS	X-Ray photoelectron spectroscopy
