# Supplementary material for: Kinetics of Seawater Diffusion into an Anticorrosion Coating with Modified Carbon Nanomaterials
Source: Nanomaterials (Basel). 2026 Oct 24;16(21):1351. doi: 10.3390/nano16211351 (PMC13765461; doi:10.3390/nano16211351)
Supplement: Supplementary file 1 [file nanomaterials-16-01351-s001.zip › nanomaterials-4584921-supplementary.pdf]

Supplementary materials for paper **"Kinetics of seawater diffusion into an anticorrosion coating with modified carbon nanomaterials", Sergey V. Yakovlev, Stepan Yu. Kupreenko, Dmitry N. Stolbov [2], Anton S. Ivanov, Andrey N. Kharlanov, Konstantin I. Maslakov, Evgeniya V. Suslova, Serguei V. Savilov**

S1 Characteristics of the enamel XC-5226

Enamel XC-5226 (NTC Spetsemal LLC, St. Petersburg, Russia).

Surface dry time at 20 ± 2 °C: ≤ 3 hours

Non-volatile content (by mass): 75–79%

Application rate (per coat): 185–220 g/m²

Dry film thickness (DFT) per coat: 50–60 μm

Recommended application: 2 coats

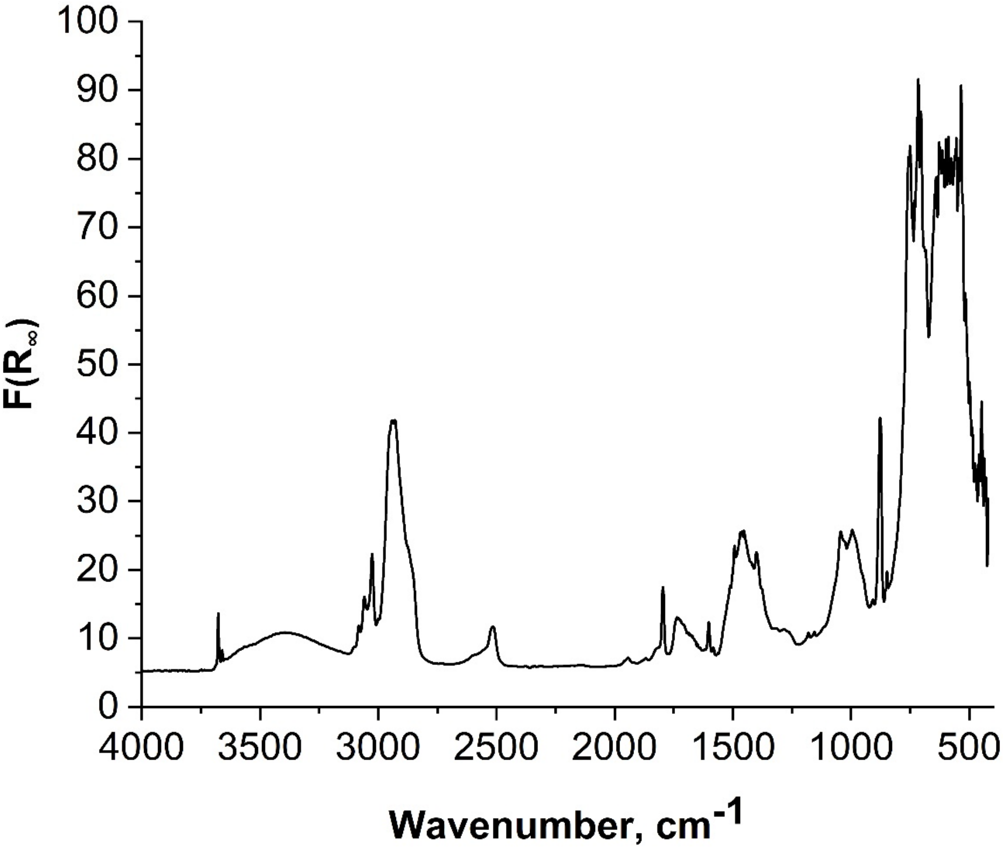

Figure S1. Diffuse reflectance infrared spectrum of Enamel XC-5226.

S2 Nyquist plots of the steel plate coated with the P-GNF-modified enamel upon immersion in 3.5% NaCl solution

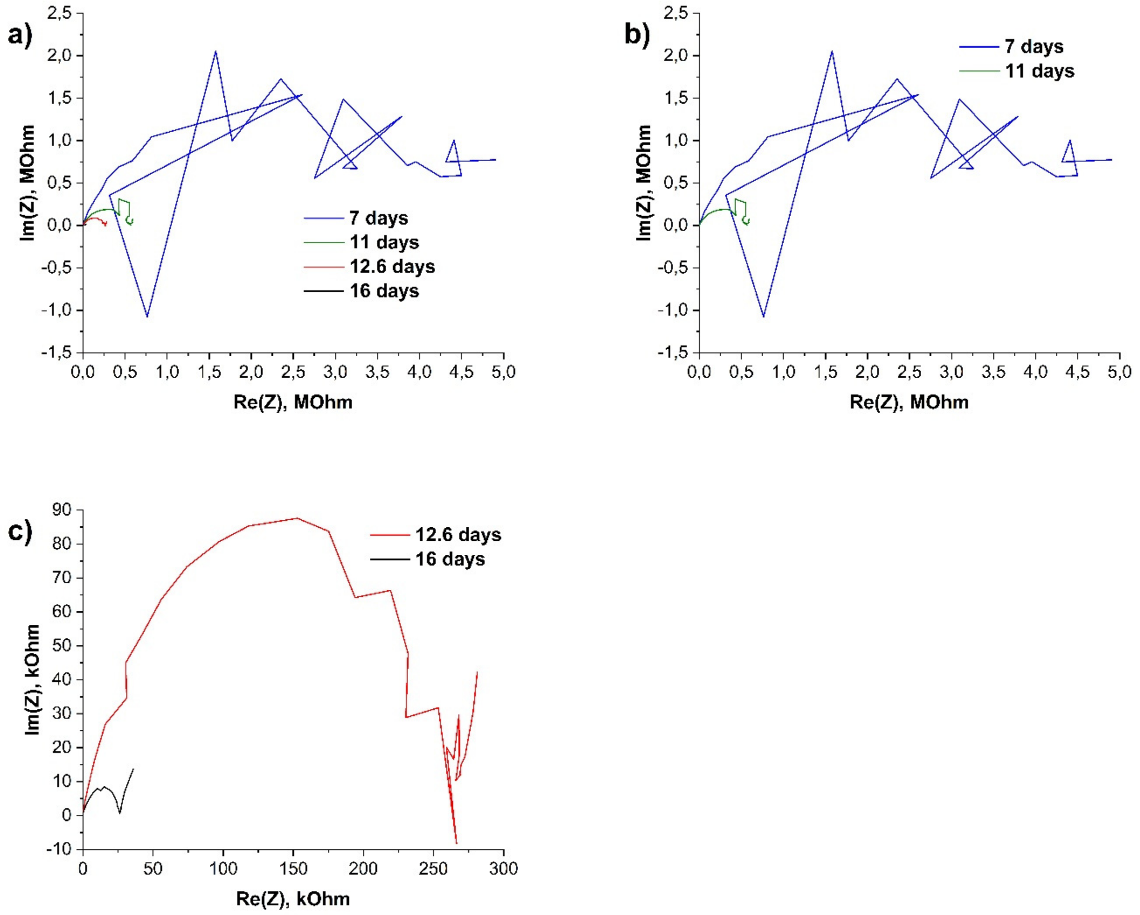

Figure S2. Nyquist plots of the steel plate coated with the P-GNF-modified enamel after different immersion times in 3.5% NaCl solution.

S3 Laser scanning confocal microscopy (LSCM) images of the coatings before and after electrochemical tests in 3.5% NaCl solution

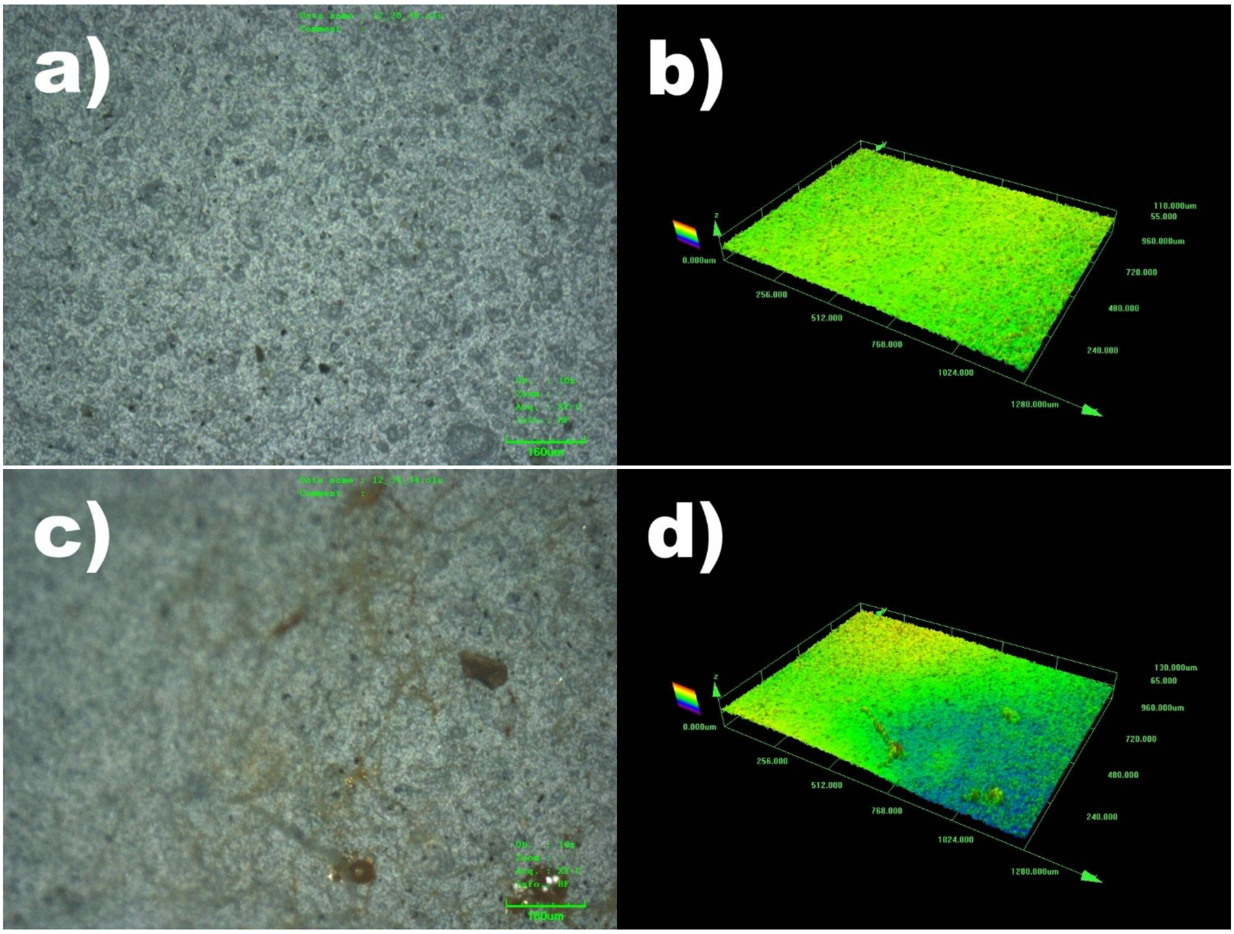

Figure S3. Optical (a, c) and 3D LSCM images (b, d) of the GNFox-modified enamel coating before (a, b) and after (c, d) electrochemical tests in 3.5% NaCl solution.

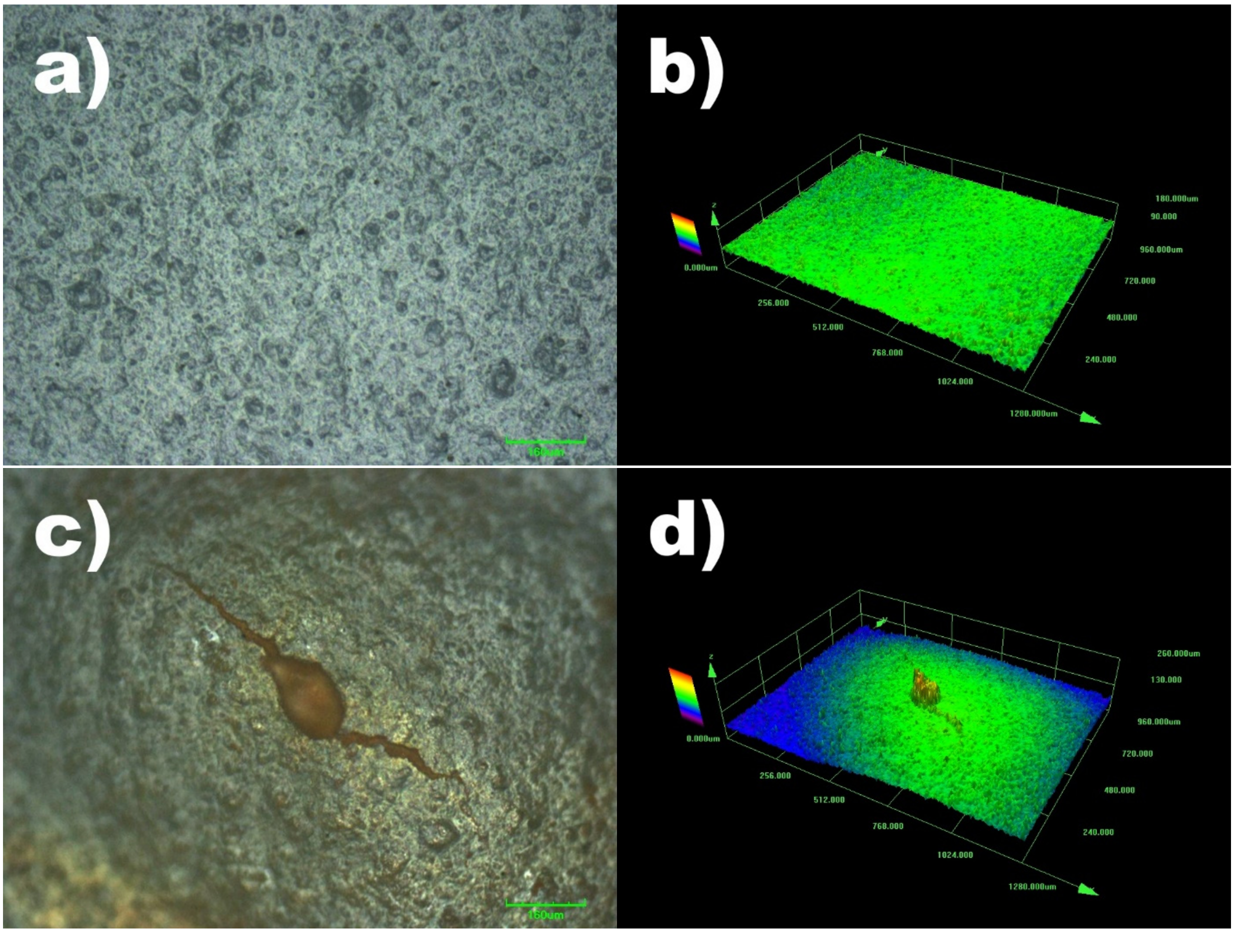

Figure S4. Optical (a, c) and 3D LSCM images (b, d) of the CNTox-modified enamel coating before (a, b) and after (c, d) electrochemical tests in 3.5% NaCl solution.

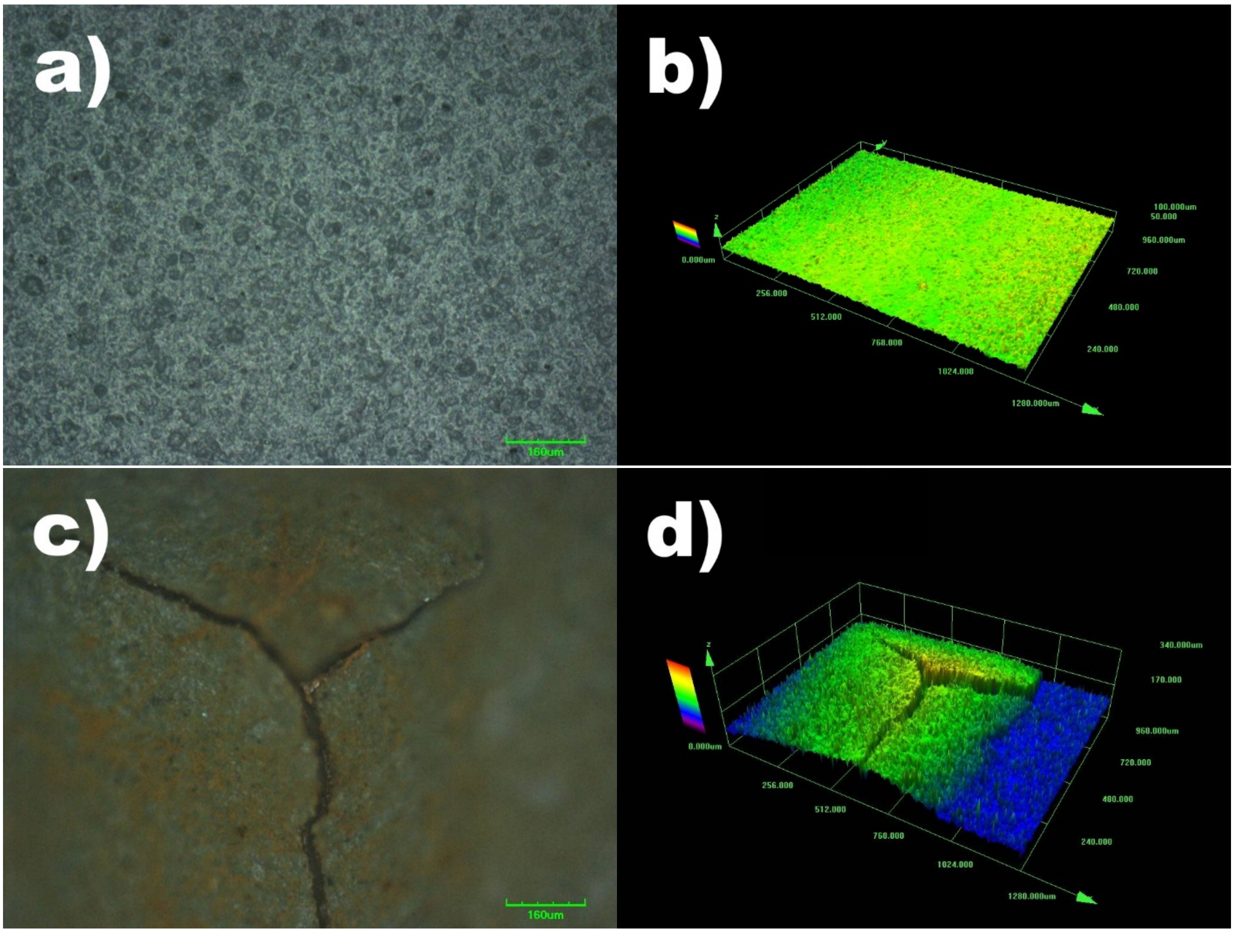

Figure S5. Optical (a, c) and 3D LSCM images (b, d) of the N-GNF-modified enamel coating before (a, b) and after (c, d) electrochemical tests in 3.5% NaCl solution.

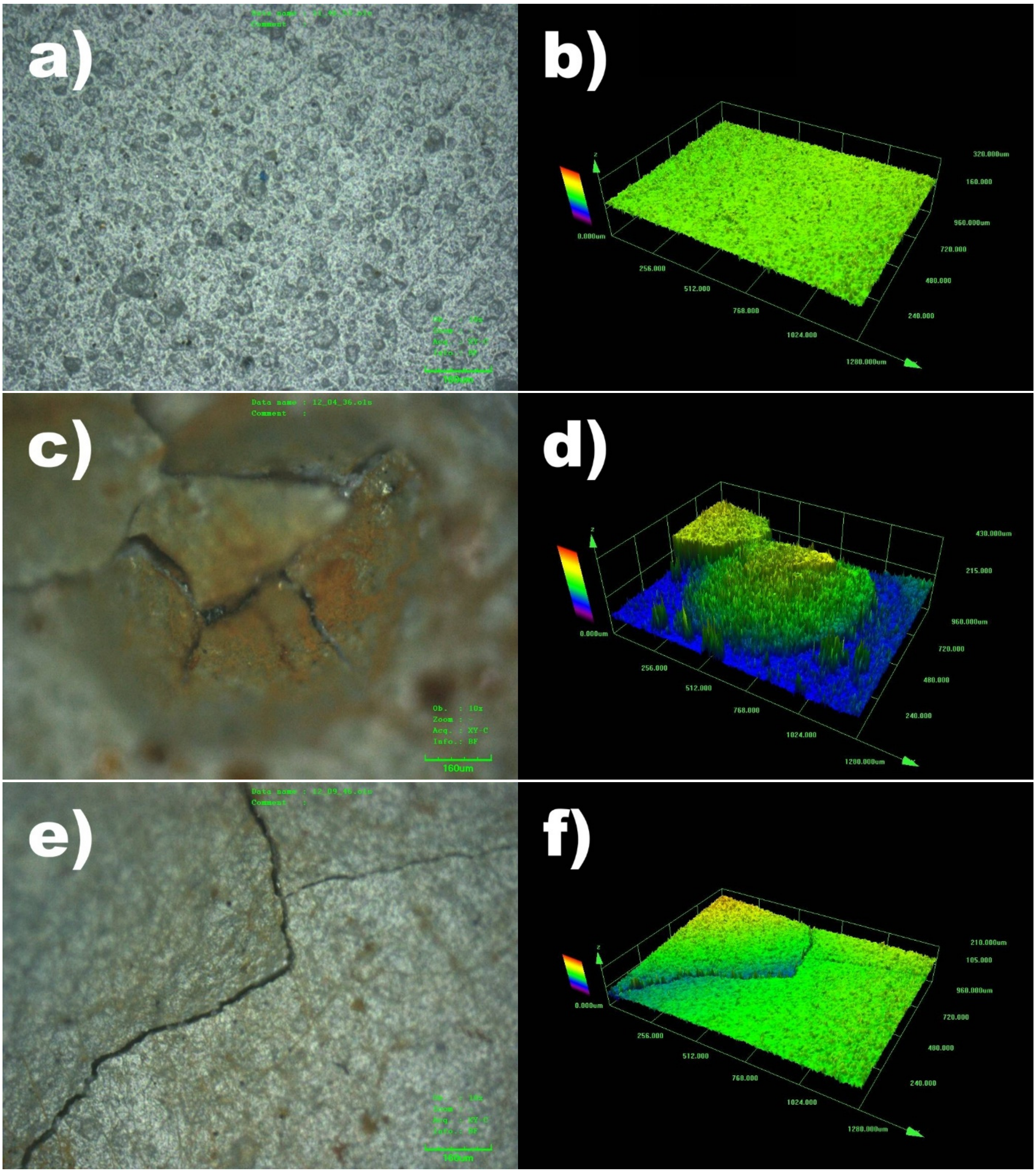

Figure S6. Optical (a, c, e) and 3D LSCM images (b, d, f) of the P-GNF -modified enamel coating before (a, b) and after (c, d, e, f) electrochemical tests in 3.5% NaCl solution.

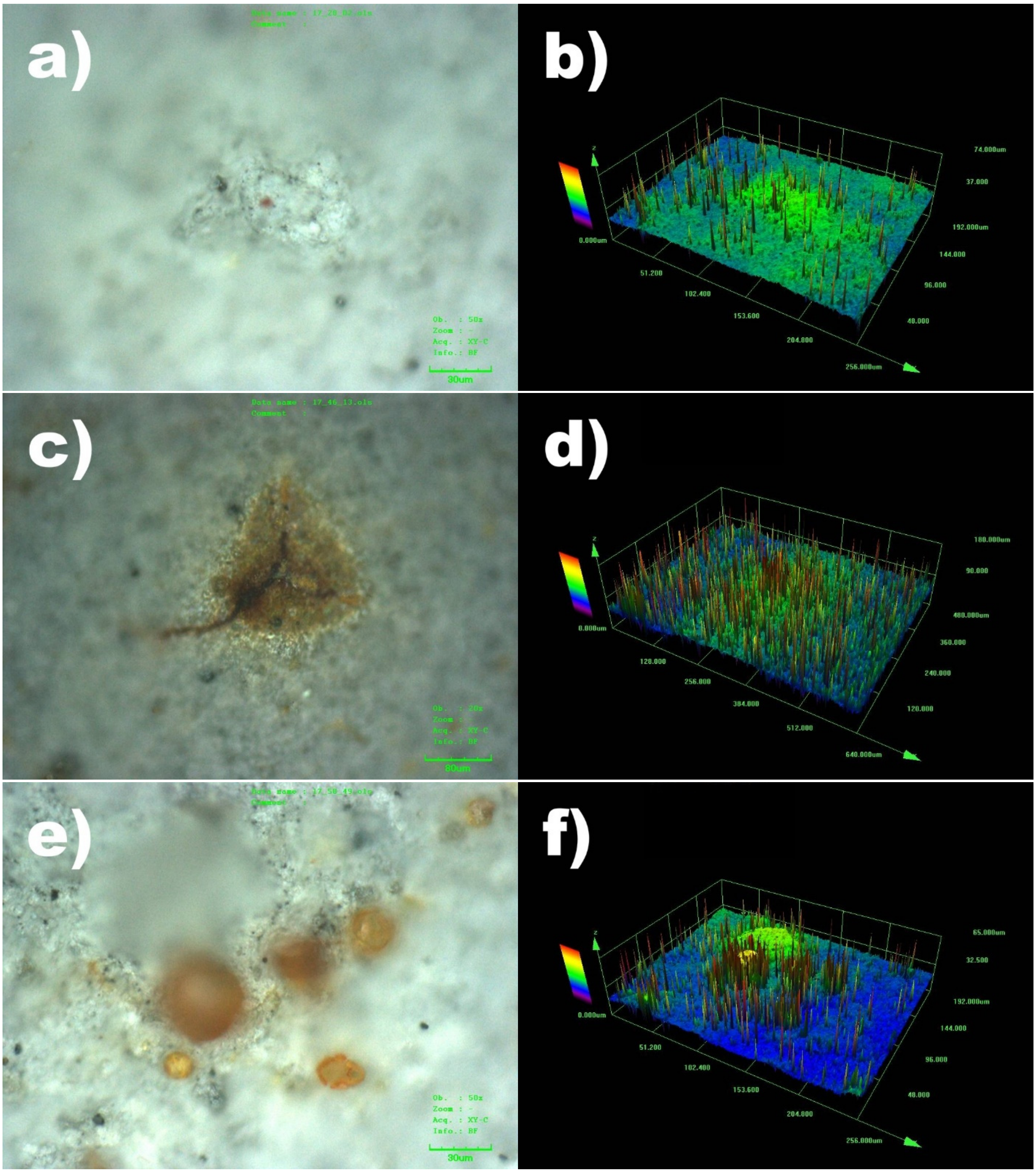

Figure S7. Optical (a, c, e) and 3D LSCM images (b, d, f) of the pristine enamel coating before (a, b) and after (c, d, e, f) electrochemical tests in 3.5% NaCl solution.

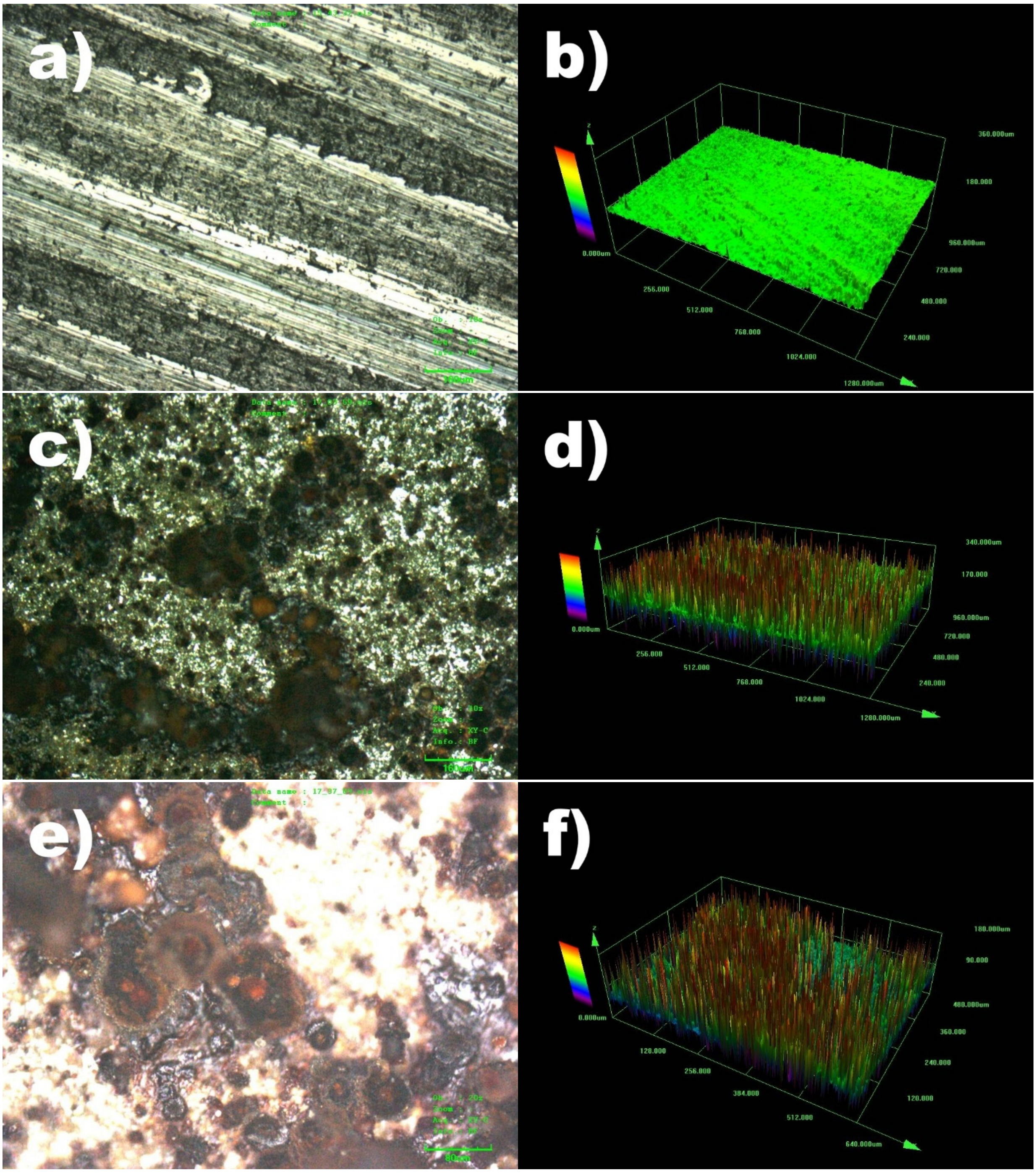

Figure S8. Optical (a, c, e) and 3D LSCM images (b, d, f) of the uncoated steel before (a, b) and after (c, d, e, f) electrochemical tests in 3.5% NaCl solution.
